# Recent Progress of Rare‐Earth Doped Upconversion Nanoparticles: Synthesis, Optimization, and Applications

**DOI:** 10.1002/advs.201901358

**Published:** 2019-09-30

**Authors:** Xiaohui Zhu, Jing Zhang, Jinliang Liu, Yong Zhang

**Affiliations:** ^1^ School of Environmental and Chemical Engineering Shanghai University 99 Shangda Road, Baoshan District Shanghai 200444 China; ^2^ Department of Biomedical Engineering Faculty of Engineering National University of Singapore Block E4 #04‐08, 4 Engineering Drive 3 Singapore 117583 Singapore

**Keywords:** NIR light, optimization, rare earth, synthesis, upconversion

## Abstract

Upconversion is a nonlinear optical phenomenon that involves the emission of high‐energy photons by sequential absorption of two or more low‐energy excitation photons. Due to their excellent physiochemical properties such as deep penetration depth, little damage to samples, and high chemical stability, upconversion nanoparticles (UCNPs) are extensively applied in bioimaging, biosensing, theranostic, and photochemical reactions. Here, recent achievements in the synthesis, optimization, and applications of UCNP‐based nanomaterials are reviewed. The state‐of‐the‐art approaches to synthesize UCNPs in the past few years are introduced first, followed by a summary of several strategies to optimize upconversion emissive properties and various applications of UCNPs. Lastly, the challenges and future perspectives of UCNPs are provided as a conclusion.

## Graphical abstract

**Due to their excellent physiochemical properties**, rare‐earth based upconversion nanoparticles are extensively applied in bioimaging, biosensing, phototherapy, optogenetics, etc. Research progress in synthesis, optimization, and various applications of rare‐earth based upconversion nanoparticles is summarized and evaluated.

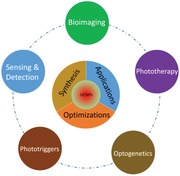

## 1 Introduction

Fluorescence refers to a process in which some susceptible atoms (molecules) that emit electromagnetic radiation (light) from the excited states caused by some external stimulus such as light absorption, mechanical action, chemical reaction, etc.^1^, ^2^, ^3^, ^4^ As one type of fluorescence, photoluminescence represents a luminescence process, where a molecule is excited by photons with a certain wavelength (e.g., ultraviolet light or visible light) and subsequently emits light with different wavelength after a short interval. In general, the photoluminescence obeys the Stokes' law that states the wavelength of the emitted fluorescence light should always be longer than that of the incident light, termed the “downconversion luminescence.”^4^ However, in some case, the emitted fluorescence light may have shorter wavelength than that of the incident light, i.e., the emitted wavelength has greater energy than the incident one, which is referred to “anti‐Stokes luminescence” or “upconversion luminescence (UCL).”^5^

The UCL scheme was first proposed by Bloembergen^5^ and later demonstrated by Porter in 1961.^6^ The UCL process refers to a nonlinear optical process, where the sequential absorption of multiple low‐energy photons through the long‐lived intermediate energy states eventually leads to emission of high‐energy photons. In recent decades, various upconversion nanoparticles (UCNPs) have been reported for upconversion luminescence including rare‐earth doped UCNPs,^7^ carbon quantum dots (QDs),^8^ graphene quantum dots,^8^, ^9^ CdSe and CdS nanoparticles,^10^, ^11^ CdSe/InP colloidal nanoparticles,^12^ PbSe/CdSe/CdS heterostructures,^13^ etc. Among these UCNPs, the rare‐earth doped UCNPs have attracted considerable attentions due to their unique physiochemical properties. For example, unlike semiconductor quantum dots whose fluorescent emissions are closely related with the particle size, the emissions of rare‐earth doped UCNPs are less affected.^14^ In addition, the electronic configuration of RE elements (rare‐earth ions) is [Xe]4f*^n^*5s^2^5p^6^ (*n* = 0–14), which indicates that the 4f electrons are greatly shielded by the outer 5s and 5p electrons and hence the electronic transitions from 4f to 4f or from 4f to 5d are barely affected by the surrounding environment. Therefore, the rare‐earth doped UCNPs have abundant energy levels and several excellent spectroscopic characters such as, long lifetime emission, narrow bandwidth, etc.^15^, ^16^, ^17^ In addition, rare‐earth doped UCNPs have exhibited higher resistance to photoblinking and photobleaching as well as narrower emission bands compared to quantum dots.^18^, ^19^, ^20^, ^21^ Because of these unique advantages, rare‐earth doped UCNPs have been tremendously exploited in bioimaging,^22^, ^23^, ^24^ biosensing,^25^, ^26^, ^27^, ^28^, ^29^, ^30^ photovoltaic devices,^31^ and photochemical reactions.^32^ Moreover, the rare‐earth doped UCNPs can typically be photo‐excited by near infrared (NIR) light. For example, the Yb^3+^ sensitized UCNPs can be excited by 980 nm laser light and Nd^3+^ sensitized UCNPs can be excited by 808 nm laser light. The use of NIR light to excite photoluminescence in UCNPs enables deeper penetration depth in biological issues and allows for noninvasive photo‐treatment. In addition, compared to UV or visible light, the NIR has much lower energy, which causes fewer damages to biosamples and generates weaker autofluorescence from biological background.

In the past few years, various new synthetic approaches and strategies have been proposed to optimize the upconversion emission including, nanoengineering of 3D shapes of UCNPs,^33^ dielectric superlensing‐mediated approach,^34^ confinement of migration of excitation energy,^35^ dye‐sensitization strategy,^36^, ^37^ etc. Meanwhile, UCNPs have also started to be applied in some new areas such as super‐resolution imaging,^38^ optogenetics,^39^ NIR image vision of mammals,^40^ and many other fields. A good understanding of these progresses in the area of UCNPs in the past few years would help to take full advantage of the upconversion phenomena and better explore new applications of UCNPs. Based on these considerations, we review recent achievements on the area of rare‐earth doped UCNPs in the past few years. The review will first summarize the state‐of‐the‐art approaches to synthesize UCNPs in Section 2, followed by several strategies to optimize and manipulate the upconversion emission performance in Section 3 and then by a summary of various applications of UCNPs in Section 4. The challenges and future perspectives of the rare‐earth doped UCNPs will be provided as a conclusion.

## 2 Synthesis

Various approaches have been proposed to synthesize rare‐earth doped UCNPs with well controlled size, shape, morphology, as well as improved luminescence efficiency. Up to now, the thermal decomposition, hydro(solvo)thermal, chemical co‐precipitation are common methods to synthesize UCNPs, which will be discussed below.

### 2.1 Thermal Decomposition

As one of the most effective methods to synthesize nanoparticles with high quality, thermal decomposition refers to a synthetic process in which metal–organic precursors are dissolved in the high‐boiling organic solvents and later decomposed at elevated temperatures.

The commonly used metal–organic precursors are rare‐earth organic compounds, such as trifluoroacetate, oleate, acetate, etc. The organic solvents to dissolve the metal–organic precursors are typically made of the mixture of oleic acid (OA), 1‐octadecence (ODE), and sometimes, oleylamine (OM). Among these organic solvents, ODE is chosen to provide a high‐temperature environment for the reaction due to its high‐boiling point (>300 °C). OA and OM are used as coordinative solvent and surfactant because of their long alkyl chains that can prevent nanocrystals from aggregating in the reaction process by capping their surfaces.

Zhang et al.^41^ first reported that the single‐crystalline and monodisperse LaF_3_ nanoplates could be fabricated via thermal decomposition of La(CF_3_COO)_3_ precursors in the presence of OA and ODE. Since then, this synthetic approach has been widely used to synthesize high‐quality UNCP crystals such as, NaYF_4_,^42^, ^43^ LiYF_4_,^43^, ^44^, ^45^ NaGdF_4_,^43^ and NaLuF_4_.^43^ In these studies, the rare‐earth trifluoroacetate, RE(CF_3_COO)_3_, was used as precursor that could provide both RE and F^−^ ions upon thermal decomposition.^14^ However, the thermolysis of trifluoroacetates produces toxic fluorinated and oxyfluorinated carbon species at high temperature, which requires an extremely ventilated reaction environment and also raises a safety concern.^46^ Later, Li and Zhang^47^ developed an efficient and facile method to synthesize β‐NaYF_4_:Yb, Er/Tm nanocrystals with controllable shape and upconversion emission. In this work, RE and F^−^ ions were provided by rare‐earth chloride (RECl_3_) and NH_4_F. Unlike previous methods, which required excessive fluoride reactants at high temperature, this new method was based on diffusion limited growth of nanocrystals. It was reported that all the fluoride reagents would turn to solid‐state nuclei at room temperature, which therefore significantly eliminated the HF gas and fluorinate species at high temperatures.

**Figure 1:**
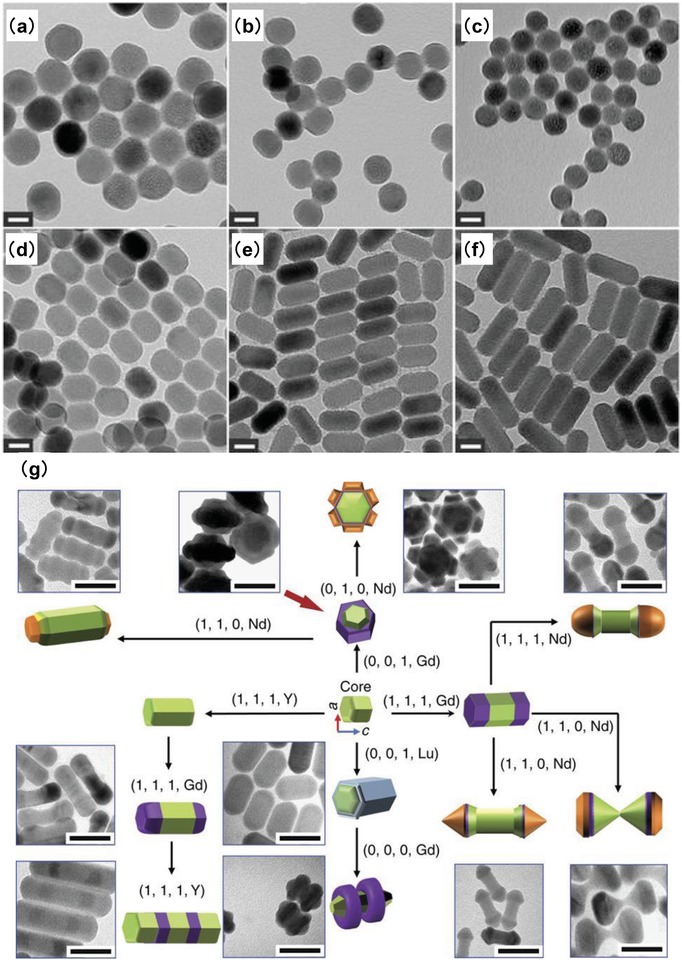
TEM images of β‐NaYF_4_:Yb, Er UCNPs synthesized at various ratios of OA to ODE of a) 2:19, b) 4:17, c) 6:15, d) 10.5:10.5, e) 15:6, f) 17:4. Reproduced with permission.^50^ Copyright 2013, Royal Society of Chemistry. g) The four digital condition codes (*R*, *T*, *F*, and RE) represent different reaction conditions where *R* = 0 and *R* = 1 represents the low and high ratio of OA^−^/OAH, respectively; *T* = 0 and *T* = 1 represents the temperature at 290 and 310 °C, respectively; *F* = 0 and *F* = 1 represents the absence and presence of an *F* ^−^ ion source, respectively; RE represents the rare‐earth ion source. By combining these different growth processes into a synthesis procedure, NaREF_4_ nanocrystals with various nanostructures can be obtained, including hourglass shaped NaYF_4_/NaGdF_4_/NaNdF_4_ nanocrystals, NaYF_4_/NaGdF_4_/NaNdF_4_ nanoflowers, NaYF_4_/NaLuF_4_ co‐axial nanocylinders, NaYF_4_/NaLuF_4_/NaGdF_4_ nanoscale spins with double rings, and NaYF_4_/NaGdF_4_/NaNdF_4_ nanodumbbells with smooth or sharp ends (scale bar, 50 nm). Reproduced with permission.^33^ Copyright 2016, Nature Publishing Group.

Recently, great efforts have been made toward a rational synthesis of UCNPs with predictable morphologies via controlled thermal decomposition by varying reaction temperature, reaction time, additive, ligand, etc.^33^, ^48^, ^49^, ^50^ For example, Na et al. once reported that the morphology of NaYF:Yb, Er/Tm UCNPs could be controlled by different OA/ODE ratios.^50^ It was reported that, with increasing the OA/ODE ratio above a certain value, the shape of obtained UCNPs changed from sphere to rod with decreased particle size and increased aspect ratio (**Figure** **1**a–f). This was because the monomers may have less opportunity to attach to the nucleated seeds when more OA molecules were added. In a very recent study, Liu et al. developed a versatile strategy to engineering various 3D shapes by programmable additive and subtractive processes.^33^ Specifically, they reported that the ratio of oleate anions (OA) to molecules (OAH) in the thermal decomposition could be used to inhibit, promote, or even etch some crystallographic facts of nanoparticles. By combing four different conditions (i.e., OA/OAH ratio, temperature, F,^−^ and rare‐earth element) into one synthesis procedure, a variety of 3D nanocrystals with core/shell/shell architecture could be obtained (Figure 1g). This was the first report of controlled fabrication of sub‐50 nm 3D heterogeneous by manipulating the growth of some crystalline facet, which allowed for engineering of new classes of heterogeneous materials with programmable shapes and functions.

Although the thermal decomposition method has become a common approach to synthesize upconversion nanoparticle with high crystallinity, uniform size and tunable morphology, it still suffers from a couple of drawbacks: 1) it usually requires high reaction temperature (>300 °C) and harsh, oxygen/water‐free reaction environment, which requires multiple vacuum‐pumping and gas purging process; 2) the nanocrystals produced in the hydrothermal decomposition method are typically capped with OA or OM, which require further surface mediation to render them water soluble.

### 2.2 Hydro(Solvo)Thermal Synthesis

Hydrothermal synthesis is another solution‐based approach, in which nanocrystals grow from the aqueous solution (e.g., water and organic solvents) in some tight and sealed reaction container at high temperature and high pressure. Taking the advantage of the high solubility and reactivity of raw materials (reactants) at high temperature and pressure, hydrothermal synthesis has become a simple and effective method to synthesize monodisperse nanoparticles with tunable morphologies and structures.

**Figure 2:**
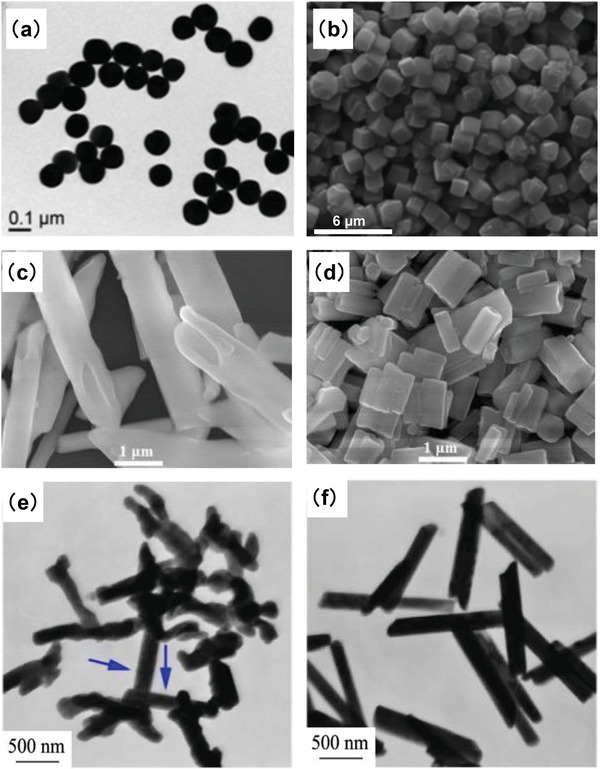
a) TEM and b) FESEM image of BaYF_5_ with RE^3+^/EDTA of a) 1:8 and b) 1:10, respectively. Reproduced with permission.^51^ Copyright 2011, Royal Society of Chemistry. c,d) FESEM image of NaYF_4_ using NaF as fluoride source at pH = 3 and pH = 5, respectively. Reproduced with permission.^52^ Copyright 2015, Elsevier. e,f) TEM image of β‐NaYF_4_:Yb^3+^, Er^3+^ nanocrystals synthesized with 1.00 and 1.25 g NaOH, respectively. Reproduced with permission.^53^ Copyright 2017, Elsevier.

In a typical hydrothermal process, rare‐earth species such as rare‐earth chloride, nitrate, and acetate are used as RE source. Various fluorides such as HF, NH_4_F, NaF, NH_4_HF_2_, NaBF_4_, and HF are employed as fluoride precursor to synthesize REF_3_ or MREF_4_ (M = alkali metal) compounds. As one of the most convenient approaches to synthesize nanocrystals, many experimental parameters can be tuned to grow nanoparticles with desired structures in hydrothermal method by varying RE/F molar ratios, pH,^51^, ^52^ fluoride precursor source,^52^ or adding OH^−^ ions^53^ or ligand agents such as citric acid,^54^ ethylene diamine tetraacetic acid (EDTA),^51^, ^55^, ^56^ cetyltrimethyl ammonium bromide (CTAB),^57^ and OA^55^, ^58^, ^59^ (**Figure** **2**).

**Figure 3:**
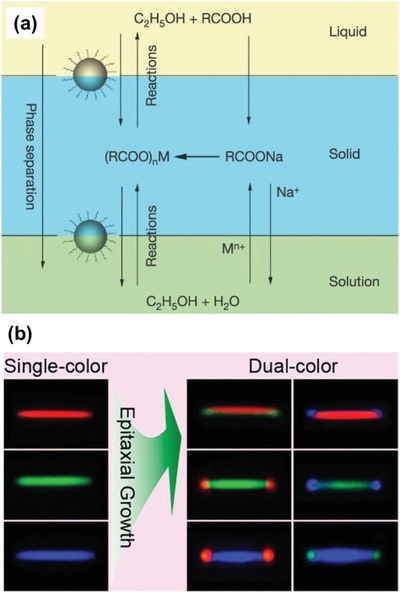
a) Scheme of liquid–solid–solution (LSS) phase transfer synthetic strategy. Reproduced with permission.^60^ Copyright 2005, Nature Publishing Group. b) Hydrothermal synthesis of dual‐color‐banded β‐NaYF_4_ microrods with different activators doped at the tips. Reproduced with permission.^67^ Copyright 2014, American Chemical Society.

Up to now, a common strategy to synthesize nanocrystals in a hydro(solvo)thermal process is the liquid–solid–solution (LSS) phase transfer and separation approach proposed by Wang et al.^60^ As shown in **Figure** **3**a, a solution of three phases is formed in the system: solid phase (sodium linoleate), liquid phase (ethanol and linoleic acid) and solution phase (water/ethanol solution containing metal ions). Taking the example of synthesizing NaYF_4_ nanocrystals based on LSS model, when the RE ions in the H_2_O/EtOH solution phase migrate to the solid phase through the ion exchange at the solid/solution phase, the RE ions and F^−^ions are co‐precipitated to form the NaYF_4_ nanoparticles. Simultaneously, due to the weight and their hydrophobic surfaces, the NaYF_4_ nanoparticles predominately stay at the bottom of the reaction container via the phase‐separation process. Using the hydrothermal approach, various rare‐earth doped nanocrystals have been synthesized, such as, NaYbF_4_,^61^NaYF_4_,^61^, ^62^ carbon‐coated NaLuF_4_,^63^ NaGdF_4_,^64^ CaF_2_,^65^ LnF_3_(Ln = La,Ce,Pr),^66^ etc. Particularly, in a recent study, Liu and co‐workers reported the hydrothermal synthesis of dual‐color‐banded β‐NaYF_4_ microrods with different activators doped at the tips^67^ (Figure 3b). It was reported that upon addition of α‐NaYF_4_ nanoparticles as the precursor in the solution of β‐NaYF_4_ microrods in the hydrothermal synthesis, the α‐NaYF_4_ nanoparticles tended to dissolve due to low thermal stability and successively grew at both ends of the β‐NaYF_4_ microrods. With this approach, a library of dual‐color‐banded upconversion microrods were prepared and could potentially be used for security inking and cell tracking applications.

### 2.3 Other Synthesis Approaches

Chemical co‐precipitation method is another friendly and convenient method to synthesize upconversion nanoparticles, which involves the precipitation of desired products out of the precursor solution. Unlike other techniques, co‐precipitation method does not require stringent reaction environment and has attracted increasing attentions. Martin et al. were the first to synthesize crystalline NaYF_4_:Yb, Pr UCNPs by a co‐precipitation technique at low temperature of 80 °C.^68^ Although this approach allowed for synthesizing nanoparticles at very low temperature, it took 24 h to obtain nanocrystals with α phase and as long as 10 days to acquire β phase. In addition, coprecipitation usually produces nanocrystals with a wide size distribution ranging from sub‐micrometers to even micrometers. Wang and Liu, also pointed out that subsequent annealing process was necessary to improve the UCNP quality after coprecipitation synthesis.^16^

Microwave‐assisted heating approach is a new developed method in which reactants are exposed to electromagnetic waves and heated up in a very short time. Compared with other techniques, microwave‐assisted heating method requires much less reaction time and fewer energy consumptions and is considered as green and promising strategy to synthesize nanocrystals. Wang and Nann once reported that monodisperse NaYF_4_ upconversion nanocrystals could be fabricated by microwave irradiation for 5 min in a closed vessel.^69^ In a later study, Wang et al., systematically studied the factors to control size and shape of AYF_4_:Yb, Er (with A = Na, Li) upconversion nanocrystals using microwave assisted method.^70^ They reported that the size of nanocrystals depended on the reaction time while the morphology was controlled by the reactant concentration and composition. With lower concentration, the resulting nanoparticles exhibited a spherical morphology but gradually changed to flower shape with increasing the concentration.

## 3 Optimization

Despite many excellent advantages over other fluorescent counterparts, UCNPs still suffer from the poor luminescence efficiency caused by many factors such as, little absorption efficiency, non‐negligible surface defects, concentration quenching, etc. Therefore, various strategies, including attaching organic dye molecules, restoring surface defects, confining energy migration, etc. have been proposed to improve the upconversion luminescence in recent years. **Table** **1** summarizes some of these common strategies to optimize upconversion performance. In addition, although cross‐relaxation has long been considered as detrimental to upconversion luminescence, new studies have shown that cross‐relaxation can also be employed as a powerful tool to manipulate the upconversion emissions. These recent advances in optimization and tailoring of upconversion are discussed in this section.

**Table 1:** Summary of various strategies to optimize upconversion performance

Strategy^a)^	Material composition	Structure	Excitation wavelength	Method and mechanism	Ref.
Dye‐sensitization	NaYF_4_:Yb/Er	C	800 nm	Efficient energy transfer via: IR 806 dye→UCNPs	71
	NaYbF4:Tm@NaYF4:Nd	C/S	800 nm	Efficient energy transfer via: IR 808 dye→UCNPs	72
	NaYF_4_:Yb/X @NaYbF_4_@NaYF_4_:Nd (X = Er, Ho, Tm, Pr)	C/S/S	800 nm	Efficient energy transfer via: ICG dye dye→UCNPs	73
	NaYF_4_:Gd/Yb/Er	C	808 nm	Efficient energy transfer via: IR 806 dye S_1_→T_1_→UCNPs	36
	NaYF_4_:Yb/Gd/Lu/Er	C	980 nm	Efficient energy transfer via: UCNP→ATTO 542 dye	37
Suppression of surface‐related quenching	NaGdF_4_:Yb/Tm@NaGdF_4_	C/S	980 nm	Epitaxial growth of NaGdF_4_ inert shell	74
	NaErF4/NaLuF_4_	C/S	980 nm	Epitaxial growth of NaLuF_4_ inert shell	75
	NaYF_4_:Tb,Tm@NaYbF_4_:Tm@NaYF_4_	C/S/S	980 nm	Epitaxial growth of NaYF_4_ inert shell	76
	KLu_2_F_7_:Yb/Er	C	980 nm	Recovery of surface defects by post‐annealing	77
Elimination of deleterious cross relaxation	NaYF_4_:Yb/Er@NaYF_4_:Yb@NaYF_4_:Yb/Er@ NaYF_4_:Yb	C/S/S/S	980 nm	Spatial separation of emitter layers	78
	NaYF_4_:Yb/Gd@ NaYF_4_:Er/Yb	C/S	980 nm	Confinement of sensitizers and emitters in different shell	79
Confinement of energy migration	NaYF_4_@NaYb F_4_:Tm@NaYF_4_	C/S/S	980 nm	Confinement of excitation energy migration	35
Construction of active‐core/active‐shell	NaGdF_4_:Yb, Er@NaGdF_4_:Yb	C/S	980 nm	Doping of Yb sensitizers in the outer shell	80
	NaGdF_4_:Yb@NaYF_4_:Yb/Er@NaGdF_4_:Yb	C/S/S	980 nm	Doping of Yb sensitizers in the core and outermost shell	81
Coupling of surface plasmon resonance	NaYF_4_:Yb, Er/Ag nanowire	C	980 nm	Enhancement of upconversion by LSPR of Ag nanowires	82
	ZrO_2_/gold nanorods	C	980 nm	Enhancement of upconversion by LSPR of Au nanorods	83
Dielectric superlensing	UCNP‐embedded PDMS substrate with PEGDA microbeads	C/S	980 nm	Amplification of upconversion mediated by dielectric superlensing	34

^a)^ Abbreviations: C, core; C/S, core/shell; C/S/S, core/shell/shell; C/S/S/S, core/shell/shell/shell; LSPR, localized surface plasmon resonance.

### 3.1 Dye‐Sensitization Strategy

**Figure 4:**
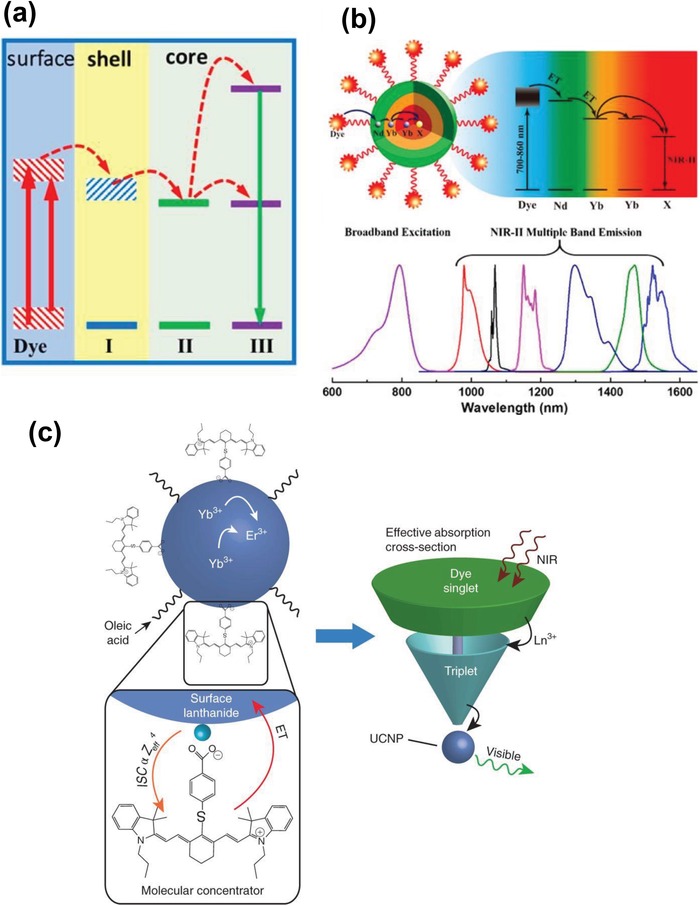
a) Proposed energy‐cascaded upconversion (ECU), involving the use of an organic dye, three types of lanthanide ions, and a core–shell design. Reproduced with permission.^72^ Copyright 2015, American Chemical Society. b) Schematic illustrations of energy transfer pathway from ICG on the surface of NaYF_4_:Yb^3+^/X^3+^@NaYbF_4_@NaYF_4_:Nd^3+^ nanocrystal, to the Nd^3+^ ions in the outer shell, then to the Yb^3+^ in the inner shell, and finally to the Yb^3+^/X^3+^ (X = null, Er, Ho, Tm, or Pr) in the core, producing large Stokes‐shifted NIR‐II emissions. Reproduced with permission.^73^ Copyright 2016, American Chemical Society. c) Cartoon schematic of the dye‐sensitized UCNP system, showing the antenna‐like nature of IR806 in sensitizing the UCNP upconversion, conveying the much larger absorption cross‐section of IR806 relative to the UCNP, as well as S_1_→T_1_ ISC enhancement by Ln^3+^. Reproduced with permission.^36^ Copyright 2018, Nature Publishing Group.

One of the intrinsic reasons for the limited practical applications of UCNPs is because of their extremely low absorption cross section and narrow absorption band in the NIR region. For example, Zou et al. reported that the extinction coefficient of the common β‐NaYF_4_:Yb/Er UCNPs at 975 nm was only 7 × 10^−5^ l g^−1^ cm^−1^, which was about 5 × 10^6^ times smaller than a commercial cyanine dye, IR‐806, at 806 nm.^71^ In light of this phenomenon, Zou et al. developed a first sensitization system in which the IR‐806 dye efficiently absorbed the 800 nm NIR photons as an antenna and then transferred the excitation energy to the ^2^F_5/2_ level of Yb^3+^ ions via the resonance energy transfer process. Due to the increased optical cross‐sections and absorption bandwidth, the emission of dye sensitized β‐NaYF_4_:Yb/Er UCNPs was dramatically increased by about 3300 times than that of nonsensitized nanoparticles. Later, Chen et al. introduced a concept of energy‐cascaded upconversion (ECU), which consisted of an epitaxial core/shell UCNP and an NIR dye attached on the UCNP surface.^72^ The proposed ECU mechanism involved the following steps: The NIR dye first harvested the excitation light and subsequently transferred it to sensitizer (ion I) in the shell across the organic/inorganic surface, then to sensitizer (ion II) across the core/shell interface, and ultimately to the activators (ion III) in the core to achieve the upconversion luminescence (**Figure** **4**a). As the energy levels of the NIR dye and two types of sensitizers were hierarchically aligned with small energy gaps, it allowed for a maximum overlap between the donor emission and acceptor absorption and resulted in an upconversion quantum efficiency of 19%, which was about 100 times higher than that of a typical rare‐earth doped UCNPs. Shao et al. reported another hybrid organic–inorganic system based on a core/shell/shell (CSS) UCNPs with the organic indocyannie green (ICG) dye on the nanocrystal surface.^73^ With the directional energy from ICG dye via the sensitizers in the shell to the emitters in the core, this hybrid system was reported to produce a fourfold increase in the emission intensity in the NIR‐II region (1000–1700 nm) with a broadly excitable spectral range (700–860 nm) (Figure 4b). In a very recent study, Garfield et al. revealed that the spin‐triplet states in the dye were key intermediates in the energy migration process and the heavy nucleus of rare‐earth ions at the UCNP surface could further increase the intersystem cross (ISC) from singlet to triplet state within the dye.^36^ Based on this triplet manifold mechanism, Garfield et al. designed a hybrid IR_806_/NaY_0.78−_ *_x_*Gd*_x_*Yb_0.2_Er_0.02_F_4_ nanocomposite and reported that the ISC could be greatly enhanced by the spin‐orbital coupling to Gd^3+^ ions near the UCNP surface, which could enrich the triplet population and in turn caused a significant amount of energy transfer into the UCNPs (Figure 4c).^36^ According to their analysis, with 30% Gd doped into the system, the dye‐sensitized upconversion emission was increased by about 33 000 times and upconversion efficiency by 100 times compared with bare UCNPs. Recently, Wisser et al. demonstrated another facile method to improve the upconversion quantum yields in UCNPs via emission dye‐sensitization strategy.^37^ In this study, the commercially available dye ATTO 542 was attached to the surface of UCNPs (Na(Y/Gd/Lu)_0.8_F_4_:Yb_0.18_Er_0.02_). Because the ATTO 542 dye has high radiative rate and its lowest unoccupied molecular orbital (LUMO) of ATTO 542 dye is well matched with the ^2^H_11/2_ and ^4^S_3/2_ states of Er^3+^ ions, the efficient energy transfer is expected to occur from UCNPs to the dye. It was reported that the dye sensitization dramatically improved the upconversion quantum yield for UCNPs of different sizes, with the highest enhancement of ten times for the smallest particles studied (10.9 nm in diameter). The approach to enhance upconversion performance by sensitizing emission with a fluorescence dye highlighted the importance of enhancing radiative rates in rare‐earth doped UCNPs, which could inspire the design of UCNP composite with completely new architecture.

### 3.2 Minimization of Concentration Quenching

From the point of view of the microstructure of a single UCNP crystal, it usually contains thousands of photon sensitizers and hundreds of photon activators.^76^ Theoretically, increasing the concentration of the sensitizers and activators in the UCNP crystal can boost the brightness and efficiency of upconversion luminescence. However, above a certain threshold, further increase of dopant concentration can lead to luminescence quenching. Currently, two common models have been proposed to explain the origin of the concentration quenching: one is the enhanced energy migration to the surface defects^84^ and the other is deleterious cross‐relaxation between dopant ions.^85^ Due to the “concentration quenching” effect, the sensitizer concentrations, i.e., the typical trivalent Yb^3+^, are commonly around 20 mol%, and activator concentrations are generally limited to 0.5–5 mol%. In this regard, developing methodologies to alleviate the concentration quenching threshold of upconversion luminescence for sensitizer and emitter ions is critical for access to much brighter UCNPs and expanding their practical applications.

#### 3.2.1 Suppression of Surface‐Related Quenching

**Figure 5:**
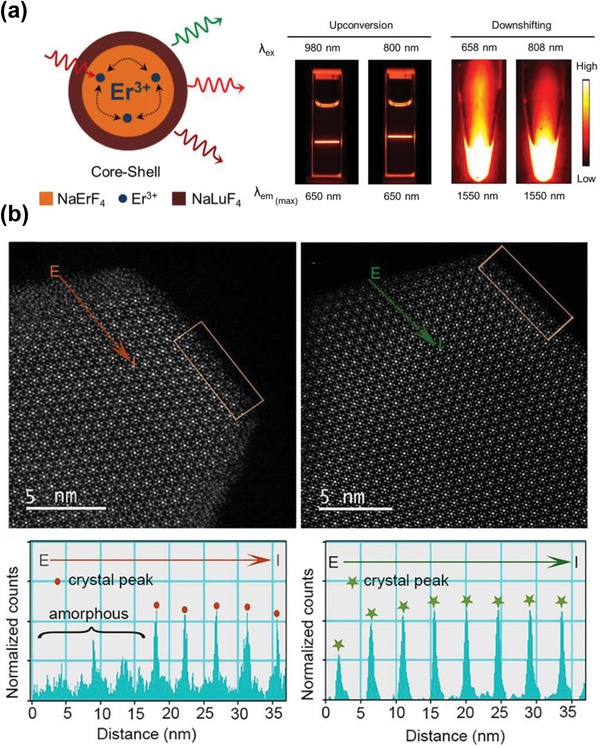
a) Schematic illustration of the NaErF_4_–NaLuF_4_ core–shell nanocrystals, which showed strong both upconversion and downshifted emission at variable excitation wavelengths. Reproduced with permission.^75^ Copyright 2017, American Chemical Society. b) HAADF‐STEM images of KLu_2_F_7_:38%Yb^3+^,2%Er^3+^ UCNPs before (top left) and after (top right) annealing at 240°C. Intensity profiles recorded by scanning along the directions of the orange (bottom left) and green arrows (bottom right) of the UCNPs before and after annealing, respectively. Reproduced with permission.^77^ Copyright 2018, American Chemical Society.

It is well established that many quenching centers are present at the surface of UCNPs, such as defects, ligands and solvent molecules with high energy vibrations,^74^, ^86^, ^87^ which can quench the excitation energy of dopant ions and lead to the reduction in luminescence intensity. Therefore, one of the most common approaches to overcome concentration quenching is to design the core–shell architecture to passivate the surface of luminescent core with an inert shell of sufficient thickness.^88^ For example, Wang et al. once reported that a remarkable increase in emission intensity of core–shell NaGdF_4_:Yb/Tm@NaGdF_4_ UCNPs compared with core only NaGdF_4_:Yb/Tm UCNPs.^74^ It was concluded that the inert NaGdF_4_ shell could efficiently protect the luminescent core from quenching centers at the surface. In a recent study, Johnson et al. demonstrated that surface passivation could also help high‐doping UCNPs confine energy migration and prevent the quenching of excitation energy at the surface.^75^ They further showed that 100 mol% Er‐doped NaYF_4_ core (i.e., NaErF_4_) with an inert NaLuF_4_ shell still exhibited enhanced emission intensity with little concentration quenching effects, for both upconverting and downconverting luminescence at different excitation wavelengths (**Figure** **5**a), suggesting that it was the energy migration to surface dominating the concentration quenching process in highly doped UCNPs. Similarly, Ma et al. reported that the optimal sensitizer concentration was limited by the surface quenching and size of crystal particles.^76^ In this study, a Tb‐rich template core was first synthesized to allow the epitaxial growth of the size‐tunable NaYbF_4_ active shell and then another inert NaYF_4_ shell was employed to passivate the UCNPs from surface defects. It was confirmed that the increase in Yb^3+^ sensitizer concentration in this sandwich design could aid in the energy sensitization and benefit the overall brightness. In addition to surface passivation by growing an inert shell, Xu and co‐workers^77^ recently proposed a wet‐chemical annealing process to restore the KLu_2_F_7_ bare core UCNPs from surface defects (i.e., vacancy, lattice disorder and interstitial defects). Using the aberration‐corrected high‐angle annular dark field scanning transmission electron microscopy(HADDF‐STEM), it was shown that the edge of the as‐grown UCNPs had an amorphous phase but post‐annealed UCNPs had a well‐defined edge with high crystallinity (Figure 5b). Due to the recovery of surface defects, the upconversion luminescence could be increased by an order of magnitude.

#### 3.2.2 Elimination of Deleterious Cross Relaxation

Another process related to concentration quenching at high dopant concentration is cross relaxation, in which the luminescent (excited) ions transfer its energy to neighboring ions and leads to the drastic reduction in luminescence intensity. Particularly, since the majority of rare‐earth ions have abundant energy states, the cross relaxation is rather more likely to occur between different types of dopants in the rare‐earth doped UCNPs. Besides, the cross relaxation are Coulomb interactions between two ions and such mutual interaction is largely dependent on the ion–ion distance.^89^ This is why the cross relaxation can be negligible at low dopant concentrations but must be taken into account at high doping concentrations when the ion–ion separation is of the order of a nm or less.^90^

One of the approaches to eliminate the detrimental effects of cross relaxation process at high doping concentrations is to spatially separate the rare‐earth ions and enlarge the dopant–dopant distance. For example, Liu et al., developed a strategy of spatial separation of the emitter doping area to alleviate the concentration quenching threshold of upconversion luminescence.^78^ In this study, the specially designed UCNPs contained two separating shells (NaYF_4_:Yb^3+^) to minimize the energy transfer between two illuminating layers (NaYF_4_:Yb, Er^3+^), thus resulting in an increase of upper threshold for concentration quenching of Er^3+^ emitters, e.g., from 2% mmol to 5% mmol. Recently, Huang et al. reported a versatile core–shell architecture, in which the high concentration of sensitizer and activator ions, i.e., Yb^3+^ and Er^3+^ions, were respectively confined in the core and shell region.^79^ Since the Yb^3+^ sensitizer ions were well isolated by the activator shell, the surface quenching effects between Yb^3+^ ions and surrounding molecules with high energy vibrational modes could be greatly suppressed. In addition, it had proven that the proposed architecture could also alleviate activator concentration quenching and could realize much higher Er^3+^ doping concentration of over 6 mmol%. Moreover, the activator Er^3+^ ions were localized in the shell region, which could further facilitate the energy transfer from UCNPs to the surface‐bound dye molecules due to the short distance between UCNP energy donors and dye acceptors.

### 3.3 Confinement of Energy Migration

**Figure 6:**
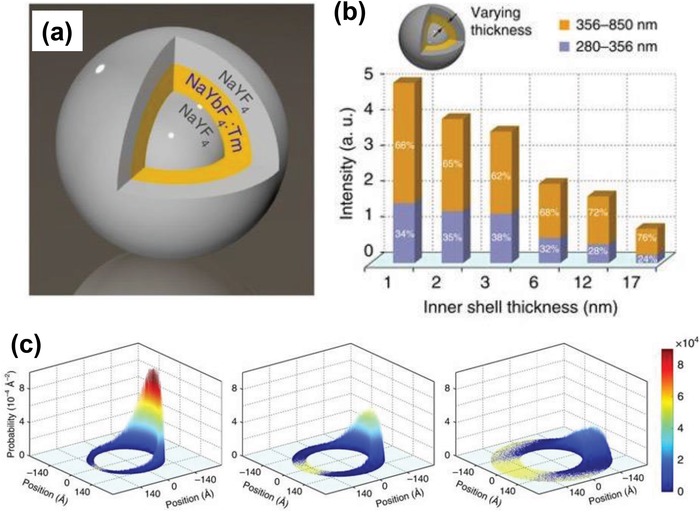
a) Schematic design of a NaYF_4_@NaYbF_4_:Tm@NaYF_4_ core–shell–shell nanoparticle for confining the migration of excitation energy generated in the Yb^3+^ ions. b) Upconversion emission intensity versus inner shell thickness (1–17 nm). c) Simulation of shell thickness on the probability distribution function of excitation energy. With increasing inner shell thickness (from left to right), the energy migrates to a larger area and the probability distribution function of excitation energy drops significantly. Reproduced with permission.^35^ Copyright 2016, Nature Publishing Group.

Manipulation of size confinement has proven to be an effective approach to construct luminescent nanomaterials with size‐tunable electronic and optical performance. Wang and co‐workers^35^ developed a methodology to tune the energy migration in NaYbF_4_ lattice by controlling the dimensions of the crystal lattice. In this work, the NaYF_4_@NaYbF_4_:Tm@NaYF_4_ core–shell–shell nanoparticles were synthesized via the layer‐by‐layer epitaxial growth process (**Figure** **6**a), along with the migration of the excitation energy confined in the NaYbF_4_ inner shell. The authors reported that the spatial confinement of excitation energy within the nanoshell could effectively avoid long distance migration of excitation energy and lower the quantity of defects accessible to the Yb sublattice. When the thickness of active NaYbF_4_ inner shell was decreased from 17 to 1 nm, the lifetime of Yb^3+^ ions were remarkably lengthened, indicating that the energy loss to the host lattice was greatly suppressed (Figure 6b). Based on the simulation of probability distribution function of excitation energy in the NaYbF_4_ shell, the energy tended to be confined in a smaller area with decreasing the inner shell thickness and reduced the possibility of coupling excitation energy to defects (Figure 6c). As a result, an efficient five‐photon upconversion emission of Tm^3+^ in the NaYbF_4_ host lattice without concentration quenching was demonstrated. In addition, a lasing emissions in deep‐ultraviolet wavelength at around 311 nm (Gd ^6^P_7/2_→^8^S_7/2_) was further achieved at high Gd^3+^ doping concentration (30 mol%) via 980 nm pulse pumping scheme, which was two orders of magnitude larger than that of a conventional Yb/Tm doped UCNP system excited at 650 nm. Through the confining energy migration strategy, Wang and co‐workers also reported that the efficient upconversion of Ce^3+^ ions could be realized in the heterogeneous core–shell–shell nanostructure of NaYbF_4_:Gd/Tm@NaGdF_4_@CaF_2_:Ce via the Gd‐mediated energy migration process(i.e., Yb^3+^→Tm^3+^→Gd^3+^→Ce^3+^), which resulted in a broad and long‐lifetime emission band in the ultraviolet region upon 980 nm laser excitation.^91^

### 3.4 Miscellaneous Strategies to Enhance Upconversion Emission

#### 3.4.1 Constructing Active‐Core/Active‐Shell Strategy

**Figure 7:**
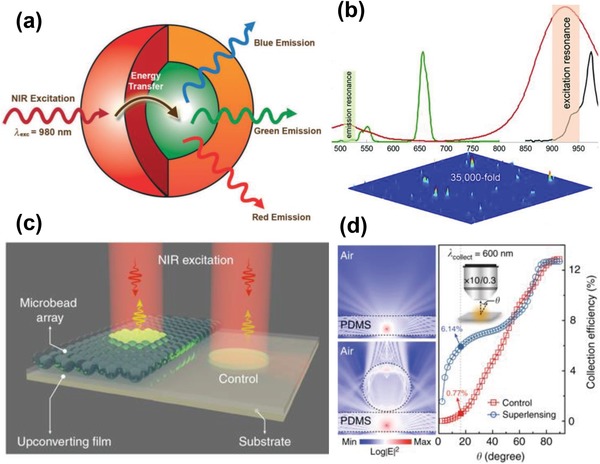
a) Schematic design active‐core/active‐shell nanoparticle architecture showing the NIR light absorbed by the active Yb^3+^‐rich shell and subsequently transferred to the luminescent core. Reproduced with permission.^80^ Copyright 2009, John Wiley & Sons, Inc. b) Cartoon schematic of the two LSPR peaks of the gold nanorods matching the excitation and emission light of ZrO_2_ UCNPs, leading to an enhancement factor of upconversion emission of ZrO_2_ UCNPs up to 35000. Reproduced with permission.^83^ Copyright 2015, John Wiley & Sons, Inc. c) Schematic illustration of the experimental setup for amplification of upconversion emission through dielectric microbeads. d) Comparison of simulation of the far‐field accumulation with and without dielectric microbeads. Panels (c) and (d) reproduced with permission.^34^ Copyright 2019, Nature Publishing Group.

As mentioned in Section 3.2, one of the most common methods to enhance upconversion emission is to grow a shell on a luminescent core, which can effectively suppress the nonradiative decay caused by the surface defects. In most cases, the shell is inert and mainly functions as a protection for luminescent ions in the core from surface defects and vibrational deactivation of the solvent and surface‐bound ligands.^80^ In recent years, a new strategy of developing active core/active shell nanoarchitecture has been proposed to enhance the upconversion emission in UCNPs. Compared with traditional active core/inert shell structure, the doped shell (usually Yb^3+^ or Nd^3+^ doped) is believed to act as sensitizers and transfers the absorbed excitation light to the luminescent core. Besides, in the active core/active shell structure, sensitizers in the core and in the shell are spatially separated, which improves the absorbance of excitation light due to higher sensitizer concentrations and reduced concentration‐dependent quenching effects. Vetrone et al.^80^ reported a strong enhancement of upconversion emissions of NaGdF_4_:Yb, Er UCNPs by coating a 20%Yb^3+^ doped NaGdF_4_ shell (i.e., “active shell”) on the core (**Figure** **7**a). It was found that the green (red) emission of the active‐core/active shell was 3 (10) times stronger than that of the active‐core/inert shell UCNPs and 13 (20) times stronger than the core‐only UCNPs. In a recent study, Ding et al. fabricated a novel NaGdF_4_:Yb@NaYF_4_:Yb/Er@NaGdF_4_:Yb sandwich‐like nanoarchitecture with the so called “active‐core/luminescent‐shell/active‐shell” configuration and reported a significant enhancement of upconversion emission due to the efficient energy transfer from Yb^3+^ sensitizers in both active core and active shell to the Er^3+^ in the middle luminescent shell.^81^ However, according to this study, excessive Yb^3+^ sensitizer concentrations would be detrimental to upconversion emissions because of the back energy transfer from the Er^3+^ to the Yb^3+^ in the core or the outer shell, and enhanced surface quenching effects caused by the migration of excitation energy within the active shell to the surface defects via Yb^3+^ ions.

#### 3.4.2 Coupling Surface Plasmon Resonance

Localized surface plasmon resonance (LSPR) is an optical phenomenon caused by the interaction of the incident light with conductive nanoparticles much smaller than the light wavelength, which leads to the plasmon oscillating locally around the nanoparticles.^92^, ^93^ Because of their excellent LSPR properties, noble metallic nanoparticles (e.g., Au and Ag nanoparticles) have been widely used to be incorporated with UCNPs to improve the upconversion emission. Feng et al. performed the first study of using Ag nanowires to enhance the upconversion emission of NaYF_4_:Yb, Er nanocrystals.^82^ According to this study, the intensity of red and green upconversion emission was enhanced by a factor of 3.7 and 2.3, respectively. Currently, a few models have been proposed to account for the mechanism of the enhancement of upconversion emissions caused by the LSPR effects of metallic nanostructures. Firstly, it is believed that the amplification of local electric field in the LSPR process may enhance the flux of incident light, as the luminous flux is proportional to the electric field, which thus promotes the NIR light absorption of UCNPs and improves the upconversion emission.^94^ In addition, when the LSPR is coupled with upconversion emission, the combination tends to increase the local density of photons and accelerates the radiative decay rates and enhances the intensity of emission.^95^ Therefore, a large LSPR‐assistant enhancement effect can be anticipated when the LSPR resonance peaks match both the excitation and emission of UCNPs. In a recent study, Zhan et al. proposed a strategy of utilizing the gold nanorods (GNRs) with two distinct LSPR peaks in order to simultaneously match both the excitation and emission light wavelength of ultrasmall (≈4 nm) ZrO_2_ UCNPs (Figure 7b).^83^ According to this study, the enhancement of upconversion emission (522 nm) of ZrO_2_ UCNP was up to 35 000‐fold when the UCNP spread its most favorable position relative to a GNR.

#### 3.4.3 Dielectric Superlensing‐Mediated Strategy

Recently, Liang et al. proposed another approach that amplified the upconversion luminescence through the modulation of dielectric microbeads.^34^ In this study, the transparent poly (ethylene glycol) diacrylate (PEG‐DA) polymeric microbeads functioned as a superlens that could efficiently confine the incident light beam into a sub‐wavelength photonic hotspot with high excitation field intensity (Figure 7c). In addition, the dielectric microbeads could simultaneously collimate the divergent upconversion emission into far‐field accumulation (Figure 7d). Through this dielectric superlensing effect, the upconversion emission could be amplified up to five orders of magnitude while the dielectric microbeads had negligible effects on the fluorescence decay lifetimes. It is believed that the dielectric superlensing‐mediated strategy would be a general solution for boosting photon–photon interactions in UCNPs and may facility the upconversion process toward practical applications under low irradiance.

### 3.5 Manipulation of Cross Relaxation

**Figure 8:**
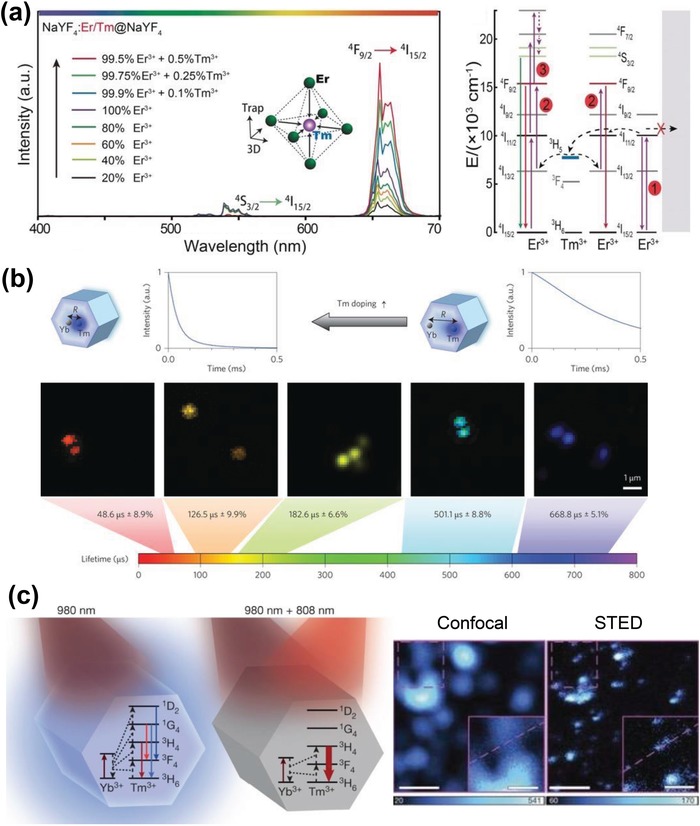
a) Upconversion emission spectra of the NaYF_4_:Er/Tm@NaYF_4_ nanoparticles doped with different Er^3+^ and Tm^3+^ concentration in the core (left) and the proposed upconversion mechanisms for NaErF_4_:Tm (0.5 mol %) nanoparticles under excitation with a 980 nm diode laser (right). Reproduced with permission.^100^ Copyright 2017, John Wiley & Sons, Inc. b) Manipulation of the luminescence decay lifetimes of NaYF_4_:Yb^3+^, Tm^3+^ UCNPs by increasing concentration of Tm^3+^. Reproduced with permission.^101^ Copyright 2014, Nature Publishing Group. c) Diagrams of energy levels of highly Tm^3+^ doped UCNPs under 980 nm and under both 980 and 808 nm laser illumination (left) and comparison of Confocal and STED image of the 13 nm 8% Tm‐doped UCNPs (right). Reproduced with permission.^38^ Copyright 2017, Nature Publishing Group.

Considering RE ions typically have abundant energy levels and various energy transfer pathways, introducing extraneous energy levels has proven to be an effective approach to manipulate the upconversion output. Chen et al. reported the first study on tuning upconversion emission from green to red by doping high concentration Ce^3+^ (>10 mol%) into the NaYF_4_:Yb/Ho UCNPs via cross‐relaxation processes between Ho^3+^ and Ce^3+^ ions.^96^ The similar strategy was also used to enhance the red upconversion emission by doping Ce^3+^ into NaY(Gd_0_._4_)F_4_:Yb/Ho UCNPs.^97^ In addition, other transition metal ions such as Mn^2+^ and Fe^3+^ have also been used to manipulate the upconversion emissions. For example, Tian et al. developed a novel Mn^2+^‐doping strategy to simultaneously control the phase, size and fluorescent behavior (red to green) of NaYF_4_:Yb/Er nanoparticles.^98^ It was reported that the Mn^2+^ doping could change the transition possibilities of Er^3+^ via energy transfer between Mn^2+^ and Er^3+^ and significantly promote the transition of red emissions. When the Mn^2+^ dopant concentration reached 30 mol%, the red to green intensity ratio was increased by a factor of ≈200 such that the color emission could be fine‐tuned from green to monochromatic red. Zhang et al. reported that doping Fe^3+^ into the β‐NaYbF_4_:Tm nanocrystals could result in enhanced ultraviolet luminescence due to the energy transfer from Yb^3+^‐Fe^3+^ dimer to Tm^3+^.^99^ In a very recent study, Chen et al. demonstrated that the appropriate doping of Tm^3+^ into Er^3+^ sensitized β‐NaErF_4_@NaYF_4_ nanocrystals could lead to a bright red upconversion luminescence because Tm^3+^ doping were thought to cause a local realignment of 4f/5d orbitals of Er^3+^ that facilitated the energy trapping and thus minimized the luminescence quenching effects (**Figure** **8**a).^100^

In addition to emission tuning, cross relaxation has also been employed to manipulate the luminescence decay lifetimes of UCNPs. Jin and co‐workers presented a new concept of lifetime multiplexing through controlling the degrees of cross relaxation by co‐doping sensitizer Yb^3+^ and emitter Tm^3+^ at different concentrations in the NaYF_4_ host nanocrystals.^101^ At different concentrations, the energy transfer from Yb^3+^ sensitizers to Tm^3+^ emitters would change due to different Yb^3+^‐Tm^3+^ distances, thus allowing for a tunability of lifetimes. Specifically, the lifetime of NaYF_4_:Yb/Tm UCNPs in the blue emission band could be gradually tuned from 48 µs (4 mol%Tm) to 668 µs (0.2 mol%Tm), along with a formation of a library of τ‐dots with tunable lifetimes for optical multiplexing (Figure 8b). It showed that the set of τ‐dots carrying distinct lifetime codes could be further used for data security applications as time‐resolved detector with proper design was required to decode these lifetime‐domain barcodes.

Another appealing example of utilizing cross‐relaxation process is to achieve super‐resolution imaging by simulated emission depletion (STED) microscopy. The conventional STED fluorescence microscopy always requires high laser intensities to realize super‐resolution imaging, which may also cause the photobleaching of the fluorophores and induce phototoxicity.^102^ Recently, Jin and co‐workers discovered a photon avalanche effect in highly doped UCNPs that could be used to achieve a super‐resolution imaging at extremely low laser power.^38^ Such photon avalanche effect was reported to be only found in UCNPs with highly doped emitters ions (i.e., 8% Tm^3+^) as the cross‐relaxation process upon 980 nm laser light could result in the rapid population inversion on metastable ^3^H_4_ levels relative to the ^3^H_6_ ground levels. Therefore, when the 808 nm laser light was later turned on, which matched the upconversion transition of ^3^H_4_→^3^H_6_, an amplified stimulated emission was triggered that discharged the ^3^H_4_ intermediate states and inhibited the blue luminescence (Figure 8c). With this strategy, a spatial resolution of sub‐30 nm was achieved at a low excitation power down to 0.19 mW cm^−2^ which was two orders of magnitude over than those currently used in STED microscopy.

## 4 Applications

### 4.1 Bioimaging

Although traditional optical imaging has become a routine technique to visualize morphological details in cells and tissues, it still suffers from several limitations such as significant auto‐fluorescence from biological background, short penetration depth, and photo‐damages to biosamples.^103^, ^104^, ^105^, ^106^ One of the reasons for these limitations is that the traditional optical imaging is mainly based on downconversion scheme that typically requires the excitation of high‐energy light, i.e., visible or UV light. In this context, the NIR‐triggered upconversion fluorescent nanoparticles are considered as a potential alternative for in vitro and in vivo imaging.

Owning to its unique NIR irradiation, the auto fluorescence from cell or tissues are rather weak under this low energy light excitation such that the UCNPs exhibit bioimaging with low optical background noise and high signal‐to‐noise (S/N) ratio. In addition, UCNP imaging exhibits large anti‐Stokes shift, narrow band emission, long lifetime emission and excellent penetration depth, which makes the UCNPs a remarkable candidate for bioimaging applications. As a result, there is a great amount of studies on using UCNPs for bioimaging both in vitro^107^, ^108^, ^109^, ^110^ and in vivo.^111^, ^112^, ^113^, ^114^ For example, Li et al. have developed a core–shell structured NaYF_4_:Yb, Er nanosphere with uniform SiO_2_ coating and successfully applied it as fluorescent probes in cell imaging.^107^ They reported that a strong fluorescence signal was observed in the MCF‐7 cells with 980 nm NIR laser irradiation after SiO_2_/NaYF_4_:Yb, Er nanospheres were incubated with cells for 24 h. In addition, with increasing the output power of the laser, only the fluorescence signal increased accordingly while the background signal from biological cells still kept constant. Chatterjee et al. developed a upconversion fluorophore consisting of NaYF_4_:Yb, Er nanoparticles and polyethyleneimine (PEI) coating and reported the first demonstration of using PEI/NaYF_4_:Yb, Er fluorophore for imaging tissues in small mammals.^111^ They showed that PEI/NaYF_4_:Yb, Er nanoparticles excited by 980 nm NIR laser exhibited much stronger fluorescence than QDs excited by UV light. They further claimed that the fluorescence emission from PEI/NaYF_4_:Yb, Er fluorophores could be even detected from a depth of 10 mm after the fluorescence particles were injected into tissues deep in the body of Wistar rat.

During the past few decades, a variety of other imaging systems that can provide anatomical and molecular information have emerged and some of them are already in clinical and preclinical use, such as, magnetic resonance imaging (MRI), computed tomography (CT), ultrasound, positron emission tomography (PET), and single photon emission computed tomography (SPECT).^115^ However, it should be notable that each of these imaging techniques has its inherent advantages and limitations when applied to different biosamples. **Table** **2** summarizes the relative benefits and limitations of common imaging techniques.^115^ For example, MRI has high sensitivity and unlimited tissue penetration depth but it suffers from long acquisition time. Optical imaging has several advantages including high sensitivity, real‐time imaging, and convenient use but it has relatively low depth penetration and limited clinical translation. PET has high sensitivity with unlimited depth penetration but it still has several disadvantages, such as high operating cost and long data collection process.^116^

**Table 2:** Summary of the relative benefits and limitations of common imaging techniques

Technique	Mechanism	Imaging contrast	Spatial resolution	Time	Depth
MRI	Electromagnetic	Paramagnetic chelates, magnetic particles	10–100 µm	Minutes to hours	No limit
CT	X‐ray	Iodinated molecules	50 µm	Minutes	No limit
Ultrasound	High‐frequency sound wave	Microbubbles	50 µm	Seconds to minutes	cm
PET	High‐energy γ rays	^18^F‐, ^14^C‐labeled compounds	1–2 mm	Minutes to hours	No limit
SPECT	Low‐energy γ rays	^99m^Tc‐, ^153^Sm‐labeled compounds	1–2 mm	Minutes to hours	No limit
Standard fluorescence imaging	Visible/NIR light	Conventional fluorescence probes	250 nm	Seconds to minutes	1–2 mm

**Figure 9:**
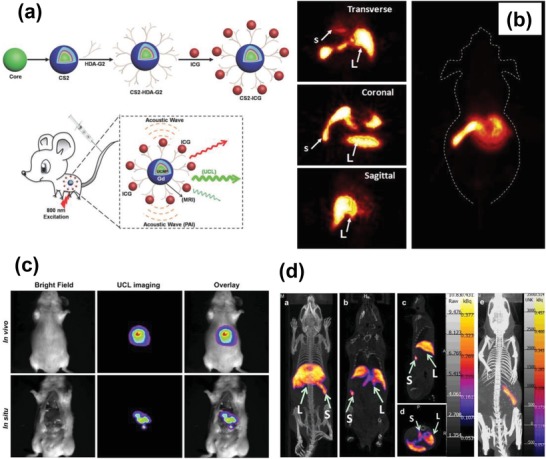
a) Scheme of deep animal imaging using high‐efficiency multi‐shell UCNPs for in vivo PAI, UCL, and MRI. Reproduced with permission.^120^ Copyright 2016, John Wiley & Sons, Inc. b) In vivo micro‐PET images of mouse acquired at 5 min after intravenous injection ^18^F‐labeled UCNPs. Reproduced with permission.^121^ Copyright 2011, Elsevier. c) In vivo UCL and d) SPECT imaging after intravenous injection of Sm‐UCNPs. Reproduced with permission.^122^ Copyright 2013, Elsevier.

Recently, great efforts have been made to develop multimodality bioimaging approach, which combines various imaging modalities into one single nanosystem and offers synergistic advantages over any modality alone.^117^ In this regard, the rare‐earth doped UCNPs are promising to achieve multimodality bioimaging as they can be tailored and engineered as functional probes for magnetism, X‐ray attenuating, radioactivity, etc.^118^ For example, He et al. developed a novel multifunctional cancer therapy platform composed of β‐NaGdF_4_:Yb/Er@β‐NaGdF_4_:Yb@β‐NaNdF_4_:Yb@MS‐Au_25_‐PEG for simultaneous PT/PA/UCL/MR/CT bioimaging.^119^ In this system, the Au_25_ shell exhibited considerable photothermal effects and offered the PT and PA properties while the Gd^3+^ and Yb^3+^ ions in UCNPs provided MR and CT imaging capabilities. In addition to multimode imaging, due to the photothermal and photodynamic effects, the fabricated multifunctional therapy platform was also reported to inhibit in vivo tumor growth under excitation of 808 nm light, which made it a promising tool to realize image‐guided cancer therapy treatments. Liu et al. designed NaYF_4_:Yb/Er @NaYF_4_:Yb@NaNdF_4_:Yb@NaYF_4_@NaGdF_4_ UCNPs with indocyanine green (ICG) dye attached on the surface, which could afford photoacoustic (PA), fluorescence, and MR multimodal imaging capabilities^120^ (**Figure** **9**a). PET and SPET are two nuclear imaging techniques which can offer ultrahigh sensitive and sub‐picomolar detection limits.^118^ PET technique requires the positron‐emitting radioactive isotopes that can give off gamma rays for imaging when the positron encounters the electrons. Among all the radionuclides, ^18^F is one of the most widely used radiotracers for PET imaging. Li and co‐workers developed a simple and efficient strategy for synthesizing ^18^F‐labeled UCNPs through a facile inorganic reaction between rare‐earth ions and F^−^ ions. It was shown that the ^18^F‐labeled UCNPs could be effectively used for PET imaging and lymph monitoring (Figure 9b).^121^ In another work, Li and co‐workers prepared a UCL and SPECT dual‐modal bioimaging system composed of NaLuF_4_:^153^Sm,Yb,Tm by introducing small amount of radioactive ^153^Sm^3+^ ions (Figure 9c,d).^122^ With the combination of UCL and SPECT imaging in vivo, the ^153^Sm‐labeled NaLuF_4_:Yb/Tm UCNPs was believed to serve as a platform for next‐generation probes for ultrasensitive molecular imaging.

### 4.2 Therapeutic Applications

#### 4.2.1 Photodynamic Therapy

**Figure 10:**
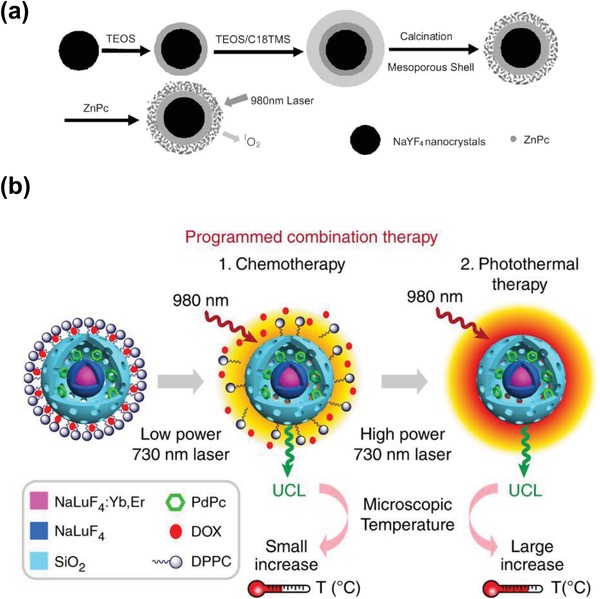
a) Schematic image showing how to load ZnPc into mesoporous silica shell on NaYF_4_@silica nanoparticles and use them for PDT. Reproduced with permission.^123^ Copyright 2009, John Wiley & Sons, Inc. b) Schematic diagram of programmed combination therapy. Reproduced with permission.^126^ Copyright 2018, Nature Publishing Group.

Photodynamic therapy (PDT) is one form of phototherapy to treat cancer cells in which some photosensitizer or photosensitizing drug is delivered into the desired/affected area such as cancer cell regions. When the photosensitizers are exposed to the light with a certain wavelength, they generate reactive oxygen species (ROS) and cause oxidative damage to surrounding cells. Compared with ultraviolet and visible light, NIR light has remarkable advantages to penetrate the tissues of the body, which also makes the UCNPs an ideal candidate for PDT applications in cancer treatment in deep tissues. Qian et al. constructed a dual functional NaYF_4_:Yb, Er@mesoporous SiO_2_ core/shell UCNPs loaded with ZnPc photosensitizers (**Figure** **10**a), which were used both in fluorescence imaging and PDT treatment.^123^ Upon 980 nm excitation, the red emission from the NaYF_4_:Yb, Er UCNPs was transferred to the ZnPc photosensitizers and triggered the PDT process. The mesoporous SiO_2_ coating avoided the direct contact between NaYF_4_:Yb, Er nanoparticles and the cell environment and improved the stability of UCNPs under harsh biological environment. In addition, the large surface area and porous structure of mesoporous SiO_2_ coating shell not only allowed more ZnPc photosensitizers stored in the shell but also facilitated the release of the generated reactive oxygen species. Later, Idris et al. developed a dual photosensitizer approach with single‐wavelength activation using UCNs and applied it in the enhanced in vivo PDT treatment for the first time.^124^ In this study, two photosensitizers, merocyanine 540 (MC540) and zinc (II) phthalocyanine (ZnPc), were loaded into the NaYF_4_:Yb, Er@mesporous SiO_2_ composite and could be simultaneously activated by the red and green emission from the NaYF_4_:Yb, Er UCNPs upon 980 laser excitation. The in vivo studies also showed that the tumor growth was greatly inhibited in the PDT‐treated mice only using 980 NIR laser irradiation, which held a great promise for the future noninvasive deep cancer therapy.

#### 4.2.2 Photothermal Therapy (PTT)

PTT is another type of phototherapy treatment that employs the photo‐induced heat for thermal ablation of cancer cells.^125^ One major application of UCNPs in a PTT therapy is to serve as an imaging probe to monitor the temperature change during the PTT process. In a very recent study, Li and co‐workers developed a temperature sensitive UCNP nanosystem for combinational chemotherapy and PTT.^126^ The system contained the photothermal agent (octabutoxyphthalocyanine palladium (II), PdPc), thermal responsive drug release unit (1,2‐dipalmitoyl‐sn‐glycero‐3‐phosphocholine, DPPC), the temperature sensor (Er^3+^ doped NaLuF_4_:Yb, Er UCNPs), and DOX drugs (Figure 10b). Upon the irradiation of 730 nm laser, the DPPC micelle would dissociate and release the pre‐encapsulated DOX drugs for mainly chemotherapy treatment at a relatively low laser power while the PTT effect would be initiated at high laser power. The upconversion emission (intensity ratio of 525–545 nm) of Er doped UCNPs was used to monitor the Eigen temperature for preventing excessive amount of heat such that the PTT and chemotherapy could be well separated for combination therapy. In addition, merging PDT and PTT into one platform to achieve the synergistic effects has also been recently demonstrated. Liu and co‐workers prepared the PAA‐caped UCNPs, which were later loaded with rose bengal (RB) and IR825 dye for performing both PDT and PTT simultaneously.^127^ In a recent study, Chan et al. prepared the NaYF_4_:Yb^3+^/Er^3+^@NaYF_4_:Yb^3+^/Nd^3+^@NaYF_4_ UCNPs, where the mesoporous silica shell‐coated gold nanorods (AuNR@mSiO_2_) were assembled via the electrostatic adsorption process.^128^ The surface plasma peaks of AuNRs were tuned to match the 541 and 654 nm fluorescence emission released by Er^3+^‐doped UCNPs, which induced the AuNRs to generate PTT effects by the SPR process. Meanwhile, the photosensitizer, merocyanine 540 (MC540), was loaded into mesoporous silica layer and absorbed the 540 nm fluorescence emitted by the UCNPs to generate ROS for PDT.

#### 4.2.3 Chemotherapy

**Figure 11:**
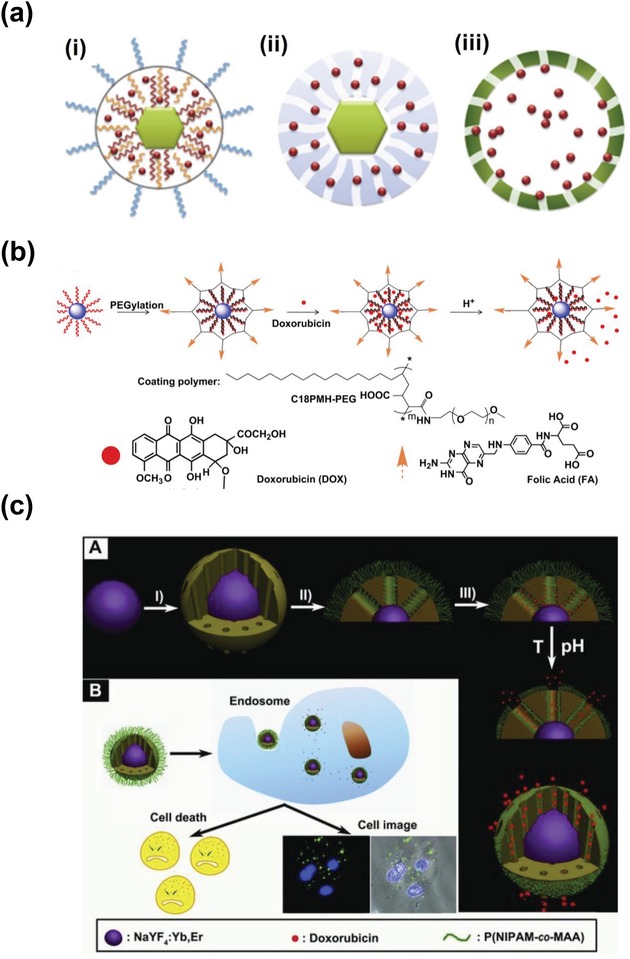
a) Schematic representation of current approaches to construct UCNP based DDSs: i) hydrophobic pockets, ii) mesoporous silica shells, and iii) hollow mesoporous‐coated spheres. Reproduced with permission.^135^ Copyright 2012, Elsevier. b) Schematic illustration of the UCNP‐based drug delivery system. DOX molecules were physically adsorbed into the oleic acid layer on the nanoparticle surface by hydrophobic and released from UCNP triggered by decreasing pH. Reproduced with permission.^134^ Copyright 2011, Elsevier. c) Synthetic route to UCNPs@mSiO_2_‐P(NIPAm‐*co*‐MAA). With increasing the temperature and decreasing the pH value, the anti‐cancer drugs would be released to cause cell death. Reproduced with permission.^136^ Copyright 2013, John Wiley & Sons, Inc.

Chemotherapy refers to a therapeutic treatment that utilizes one or more chemical drugs to treat cancer cells. Although chemotherapy has become one of the most common and effective approaches to treat cancer, it still suffers several limitations such as poor targeting efficiency, drug dependence, drug resistivity, etc.^129^, ^130^ Therefore, there is an urgent need to develop a more efficient and safe drug‐delivery system to improve the performance of conventional chemotherapy. Recently, UCNPs used as drug carriers have been extensively investigated because they have unique physicochemical properties and their size, upconversion luminescence, and surface chemistry can be easily tailored to drug‐delivery applications in vitro and in vivo.^131^, ^132^, ^133^, ^134^ Generally, there are three strategies to design the UCNP‐based drug delivery system: 1) UCNPs capped with polymer, 2) UCNPs decorated with mesoporous SiO_2_, and 3) UCNPs encapsulated by hollow mesoporous‐coated spheres (**Figure** **11**a), which have been summarized by Shen et al. in a previous review.^135^ For example, Wang et al. firstly functionalized the UCNPs with polyethylene glycol (PEG) grafted amphiphilic polymer, which was then loaded with anti‐cancer drug, doxorubicin (DOX), by physical adsorption via hydrophobic interactions (Figure 11b).^134^ It was found that the release of DOX was controlled by varying pH with an increased drug dissociate rate in slightly acidic environment, which allowed for efficient drug release in tumor cells. Recently, Zhang et al. developed another multifunctional nanocomposite composed of UCNPs and thermo/pH sensitive polymer (P(NIPAm‐co‐MAA)) gated mesoporous silica shell, in which the UCNPs were utilized as optical nanoprobe and the (P(NIPAm‐co‐MAA)) brushes were used as the “valve” to control the diffusion of embedded drugs through the porous structure of the silica shell (Figure 11c).^136^ Due to its excellent thermo/pH sensitivity, the as‐synthesized drug delivery system showed a low level of release of anti‐cancer drug at low temperature/high pH but dramatic increase of drug release at high temperature/low pH, which was favorable for the thermo/pH regulated “on‐off” drug release.

#### 4.2.4 Radiotherapy (RT)

RT is a noninvasive clinical treatment that utilizes radiation to damage cancer cells and slow tumor growth or spread in the body. In the radiotherapy treatment, the high‐energy therapeutic radiation, such as X‐rays, gamma rays or protons, is delivered on a specific lesion area without harming nearby healthy tissues. Even radiotherapy is currently employed in about 50% of cancer treatments,^137^ it still suffers from two major challenges:^138^ one is the localization of tumor region cannot be accurately identified; the other is the cancer cells are radioresistant due to complicated microenvironment (e.g., hypoxic) in tumor region.^139^ Therefore, although an adequate radiation dose destroys any tumor cells, the potential adverse effects on the normal tissues in the path of the radiation beam still limits the use of high radiotherapeutic dose in the treatment.^139^

It is well established that elements of high atomic number (*Z*) can strongly absorb the ionizing radiation.^140^ Therefore, if the high‐*Z* materials are preferentially delivered to the tumor cells, they can selectively increase the radiation dose to the tumors and lead to a much higher dose to the lesion regions compared with healthy ones.^139^ In this context, rare‐earth doped UCNPs which contain high‐*Z* elements have emerged as promising agents to improve radiotherapy efficiency of hypoxic cells.^141^ For example, Shi and co‐workers reported a versatile UCNP‐based mesoporous silica nanotheranostics which could be further loaded with hematoporphyrin (HP) sensitizer for radiotherapy and photodynamic therapy and docetaxel (Dtxl) for chemotherapy and radiotherapy.^142^ In this nanotheranostic system, the Gd doped UCNPs are used as contrasting agents in magnetic/upconversion luminescent (MR/UCL) imaging and the HP and Dtxl are for synergetic chemo‐/radio‐/photodynamic therapy upon X‐ray irradiation and NIR excitation. Because of the cooperative and synergetic interactions among the three therapy modalities, it was demonstrated that the developed nanotheranostic system could completely prevent the DNA repair and permanently damage the cancer cells. As mentioned above, the radioresistance of hypoxic tumor cells to radiotherapy remains a major challenge in the cancer treatment.^143^ Therefore, one of the approaches to solve this issue is to provide exogenous oxygen (O_2_) to the tumors. In another study, Shi and co‐workers designed 2D nanocomposites based on MnO_2_ nanosheets incorporated with UCNPs.^144^ Because of the endogenous differences between tumor and normal cells (e.g., lower pH and higher H_2_O_2_ concentration), the MnO_2_ nanosheets would be decomposed into Mn^2+^ by acidic H_2_O_2_ in tumors and generate large amount of O_2_ in tumor cells. On one hand, the decomposition of MnO_2_ nanosheets could lead to the recovery of previously quenched upconversion luminescence of UCNPs for the diagnostic/monitoring purpose in the treatment. On the other hand, the O_2_ generation on hypoxic tumors could significantly improve the radiotherapy and photodynamic efficiency in deep tumors upon NIR/X‐ray irradiation. As the first report on developing 2D theranostic nanoplatform for simultaneous imaging and oxygen‐elevated synergetic therapy, this study provides access to efficient treatment of a variety of hypoxic tumors.

In conclusion, a number of research has focused on UCNP‐based theranostic systems for various therapeutic applications, most of which in principle can respond to various exogenous (temperature, light, magnetic field, etc.) or endogenous stimuli (pH, enzyme concentration, expression levels of certain chemical species, etc.) and thus selectively target the lesion cells, without affecting healthy normal cells.^145^ However, this approach requires the therapeutic platform to identify some tumor biomarkers and respond with specific physiochemical reactions, including protonation, surface chemistry change, and decomposition of encapsulating agents in order to achieve the treatment in a dosage‐controlled manner,^145^ which, in turn, makes the therapeutic rather complicated and limits the targeting efficiency. In this context, developing simpler and more efficient UCNP‐based nanocarriers that can take effect at the right place and right time may represent an attractive approach for future cancer treatment.

### 4.3 Optogenetic Stimulation

**Figure 12:**
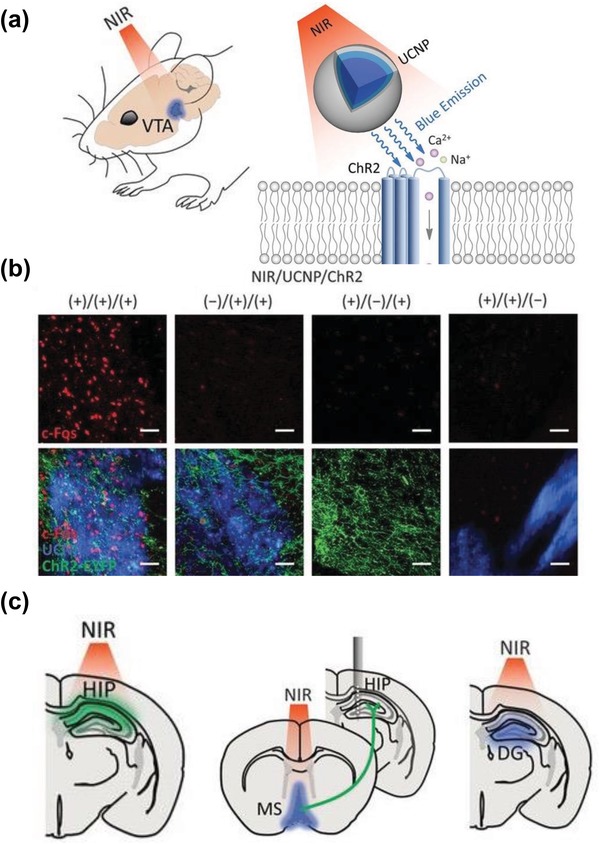
a) Cartoon schematic of UCNP‐mediated NIR upconversion optogenetics. b) Confocal images of the VTA after transcranial NIR stimulation under different treatments. Extensive NIR‐driven c‐Fos (red) expression was observed only in the presence of both UCNPs (blue) and ChR2 expression (labeled with EYFP), Scale bars: 100 µm. c) An illustration of in vivo upconversion optogenetics for multiple neural systems, including the inhibition of HIP activity, stimulation of MS, and stimulation of the hippocampal engram for memory recall. Reproduced with permission.^39^ Copyright 2018, American Association for the Advancement of Science (AAAS).

Optogenetics is a recently developed technology that can spatiotemporally stimulate genetically defined light‐sensitive ion channels and optically manipulate neural activity in the brains of behaving animals.^146^ Despite remarkable achievements, the neuronal modulations in vivo brain can only be achieved by visible wavelength window (<600 nm) which has limited tissue penetration depth. In this context, the UCNPs hold great potential for optogenetic applications as they can allow for the optogenetic stimulation in the deep brain regions with NIR light and make the optogenetics noninvasive. Zhang and co‐workers. developed a novel quasi‐continuous wave (quasi‐CW) excitation approach to improve upconversion emission of UCNPs and applied it to successfully activate the *channelrhodopsin‐2* in *Caenorhabditis elegans* , demonstrating the feasibility of utilizing UCNPs for optogenetic manipulations with NIR light.^147^ Xing and co‐workers presented a study that applied UCNPs to activate the ChR2 ion channel and manipulate Ca^2+^ influx in living cells and zebrafish upon excitation of 808 nm light.^148^ In a recent study, Chen et al. explored the possibility of using UCNPs as optogenetic actuators to non‐invasively simulate the neurons in the deep mouse brain.^39^ By tuning the upconversion emission by doping different rare‐earth ions, the NIR‐triggered upconversion optogenetics could achieve the neuronal activations as well as neuronal inhibitions (**Figure** **12**a–c). More important, it was reported that the NIR‐mediated upconversion optogenetics could silence seizure by inhibition of hippocampal excitatory cells and trigger memory recall (Figure 12c), which held great potential for remote therapy of neurological disease.

### 4.4 NIR Image Vision of Mammals

**Figure 13:**
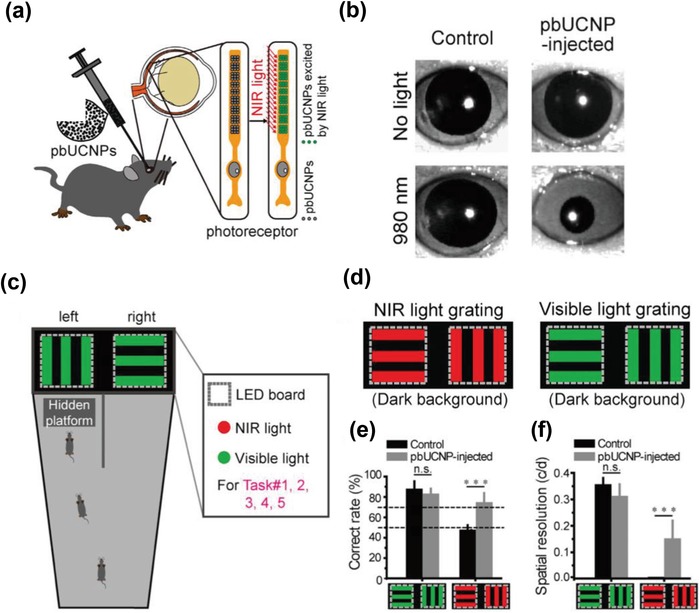
a) Illustration of sub‐retinal injection of pbUCNPs in mice. b) Images showing pupil constriction from non‐injected control and pbUCNP‐injected mice under 980 nm light stimulation. c) Diagram of Y‐shaped water maze for different tasks. d) Stimuli of one task to evaluate the mice's ability to discriminate between the two orientations (horizontal or vertical) of NIR light gratings. Experiments were under dark background. e) Correct rates of task for light grating discrimination. f) Visual spatial resolutions of pbUCNP‐injected and control mice for 535 and 980 nm light gratings. Reproduced with permission.^40^ Copyright 2019, Elsevier.

It has been long known that mammals, including humans, can only detect visible light with wavelengths ranging from 400 to 700 nm.^149^ It is still a great challenge for mammals to perceive the longer wavelength light (e.g., NIR light) through the eyes. This is mainly because the photoreceptors (rods and cones) in mammalian eyes, consisting of opsins and their covalently linked retinals, can not absorb the wavelengths longer than 700 nm such that the mammals are unable to efficiently detect the NIR light.^40^, ^150^ In a very recent study, Xue and co‐workers designed ocular injectable photoreceptor‐binding UCNPs (pbUCNPs), which could further be anchored to photoreceptors in the mouse retina.^40^ The core–shell structured UCNPs (NaYF_4_:Yb, Er@NaYF_4_) could generate green emission at 535 nm upon 980 NIR light irradiation. Therefore, after the solution of these nanoparticles was injected into the sub‐retinal spaces of lab mice, the mice would be able to see NIR irradiation as green light (**Figure** **13**a). It was reported that the pupils of the pbUCNP‐injected mice showed strong constrictions on 980 nm light illumination while the non‐injected control mice did not demonstrate the pupillary light reflex under the same illumination (Figure 13b).The NIR light sensation of pbUCNP‐injected mice was further confirmed by the Y‐shaped water maze behavioral experiments (Figure 13c), in which the mice were trained to respond to patterns with different geometries. It was found that the pbUCNP‐injected mice were able to discriminate between the vertical or horizontal grating of NIR light, whereas the non‐injected control mice made choices in the random manner (Figure 13d–f). Moreover, the implantation of pbUCNPs was reported to bestow the infrared vision on mice for up to 10 weeks and not affect the mice's ability to perceive visible light patterns at the same time. As the first report to achieve the NIR image vision in mammals, this study could not only be used to study various vision‐related behaviors of animals but also be potentially used to treat visual dysfunction in many diseases.

### 4.5 Sensing and Detection

Förster resonance energy transfer (FRET) refers to a non‐radiative energy transfer process between two adjacent molecules, i.e., the donor and acceptor, that causes a reduction in the fluorescence emission intensity of the donors and an increase in that of acceptors.^151^ Since FRET originates from the dipole–dipole interactions, the distance between the donor and acceptor is typically limited within 10 nm,^152^ which, however, is still well below the abbe diffraction limit of a common microscope. Therefore, FRET has been widely used as a valuable tool for understanding different biological and physicochemical processes such as temporal distribution of biological molecules, molecular interactions, protein–protein interactions, etc. Recently, many FRET systems in conjunction with UCNPs, i.e., UCNP–FRET platforms, have been developed and successfully applied to sense or detect various types of analytes such as pH, gas, temperature, metal ions, DNA, etc. Some of these results have been summarized in **Table** **3**.

**Table 3:** Detection results for analytes with different UCNP‐based sensors

Analyte	Sensor system	Detection range/limit detection	Example of use	Ref.
pH	NaYF_4_:Yb, Er/Bromothymol blue (BTB)	pH 6–10	Polyurethane hydrogel	153
pH	NaYF_4_:Er,Yb/neutral red(NH)	pH 6.0–7.5(+/0.06)	Human serum samples	154
pH	NaYF_4_:Yb, Er/pHrodo red	pH 2.5–7.2(+/0.3)	HeLa cells	155
O_2_	NaYF_4_:Yb, Tm/[Ir(CS)_2_ (acac)]	–	–	156
NO	NaYF_4_:Yb, Er/rhodamine B‐derived molecules (RdMs)	73 × 10^−9^ m	HeLa cells	157
CO_2_	NaYF_4_:Yb, Er/bromothymol blue (BTB)	0.11%	CO_2_ detection in argon	158
Temperature	NaYF_4_:Yb/Er	25–45 °C	HeLa cells	159
Temperature	NaYF_4_:Nd/TTA dyad	0.1 K	HeLa cells	160
Fe^3+^	NaYF_4_:Yb, Er,Tm@NaGdF_4_/Nile red derivative (NRD)	89.6 × 10^−9^ m	HeLa cells	161
Zn^2+^	PAA‐NaYF_4_:Yb/Tm@NaYF_4_/chromophores	–	HeLa cells/brain tissue/zebra fish	162
Cu^2+^	NaYF_4_:Yb, Er/Rhodamine B derivative (RBH)	0.82 × 10^−6^ m	HeLa cells	163
Cu^2+^	8‐Hydroxyquinoline‐2‐carboxylic acid (HQC)	–	HeLa cells/zebra fish	164
Ca^2+^	NaYF_4_@NaYF_4_:Yb, Er@NaYF_4_/Fluo‐4	15 × 10^−12^ m to 1.35 × 10^−6^ m	HeLa cells/mice liver tissue	165
DNA	NaYF_4_:Yb, Er/graphic oxide	5 × 10^−12^ m	–	166

#### 4.5.1 pH Sensors

**Figure 14:**
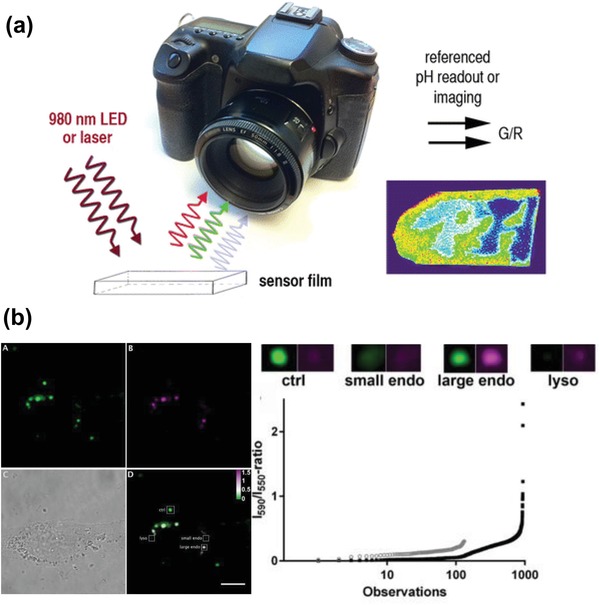
a) Illustration of sensing and imaging of the pH sensor film using the RGB technique. The sensor is excited by the IR light and emits light with different colors. The camera collects the red, green, and blue light in three independent channels, which can then be used for referenced ratiometric read‐out and imaging. pH is calculated based on rationing data from the red by the green channel data. Reproduced with permission.^154^ Copyright 2014, American Chemical Society. b) Ratiometric imaging of pH probes reveals their localization in different types of microenvironment including small endosome, large endosome, and lysosome. The intensity of ratio of *I*_590_/*I*_550_ was used to measure the pH variations in different regions of the cells. Reproduced with permission.^155^ Copyright 2017, American Chemical Society.

Sun et al. presented the first optical pH sensor, which consisted of upconverting NaYF_4_:Er,Yb nanorods and bromothymol blue (BTB) pH indicator.^153^ Depending on the pH of the aqueous solution, the BTB pH dye either exerted a strong or insignificant inner filter effect on both red and green emissions of the upconverting luminescent nanorods, which made the pH measurements achievable. Meier et al. reported a planar pH sensor based on NaYF_4_:Er,Yb upconversion fluorophore and pH indicator neutral red (NR).^154^Since the absorption peak of the pH indicator NR was spectrally matched with the green emission of the UCNPs, the green emission band of UCNPs was largely affected by the pH changes while the red emission of UCNPs remained unaffected and could serve as the referencing signal. Using this improved ratiometric approach, a great resolution of ±0.06 pH units was achieved in a pH range from 6.0 to 7.5. In addition, they further developed a convenient and straightforward method that could directly read out the pH values from a digital camera (**Figure** **14**a). Näreoja et al. developed a nanoprobe for ratiometric sensing and imaging of intracellular pH based on polyethylenimine (PEI)‐coated UCNPs.^155^ In this study, after conjugation with pH sensitive pHrodo red dye, the original UCL emission around 550 nm decreased while a new sensitized emission of the pH dye around 590 nm was observed which increased in acidic conditions. Therefore, the intensity ratio between UCL emission at 550 nm and sensitized emission of pHrodo red at 590 nm, i.e., *I*_550_/*I*_590_, could function as a good fingerprint to sense the pH changes. It is noted that the branched PEI coating used in this work has three advantages: 1) providing large number of reactive primary amino (−NH_2_) groups for a better conjugation of pH indicator dye on the UNCP surface; 2) improving the colloidal stability with a zeta potential up to 50 mV in deionized water; 3) enhancing the cellular uptake of UCNPs due to its high positive surface charge. It was demonstrated that this UCNP‐based pH nanoprobe was well suited for detecting pH changes in in vitro modes and could serve as a ratiometric sensor for studying compartmentalization and compartment acidification with confocal laser scanning microscope (Figure 14b).

#### 4.5.2 Gas Sensors

**Figure 15:**
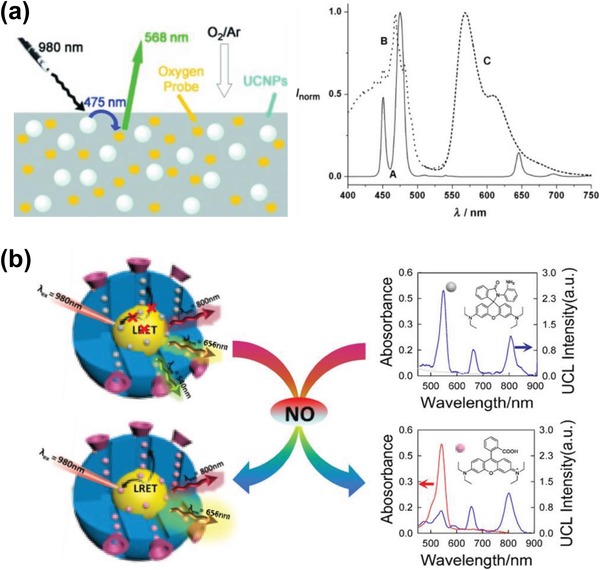
a) Schematic of the inner filter‐effect‐based UCNP O_2_ sensor. The absorbance spectrum of oxygen sensor [Ir(CS)_2_(acac)] overlapped with the emission of NaYF_4_:Yb, Tm UCNPs while the green emission of [Ir(CS)_2_(acac)] could be quenched by the O_2_. Reproduced with permission.^156^ Copyright 2011, John Wiley & Sons, Inc. b) Schematic illustration of the sensing principle of the upconversion nanoprobe for ratiometric luminescent measurement of nitric oxide. Reproduced with permission.^157^ Copyright 2017, American Chemical Society.

Gas molecules such as O_2_, NO, CO_2_, and NH_3_ are commonly considered as bioactive signaling molecules that are actively involved in various physiological behaviors such as metabolism, immune regulation, apoptosis, neural communication, etc. Therefore, sensing and imaging of these gas molecules have attracted considerable interests over the past decades. Achatz et al. first developed a nanoplatform for sensing oxygen that can be performed with NIR light.^156^ In this system, the NaYF_4_:Yb/Tm upconversion nanoparticles acted as nanolamps and gave shortwave emissions at 455 and 475 nm upon laser excitation at 980 nm to photoexcite the oxygen probe, [Ir(CS)_2_(acac)], whose absorbance had a maximum at 468 nm. The green emission of Ir(III)‐complex at around 568 nm due to the excitation at around 470 nm was strongly quenched by oxygen (**Figure** **15**a), which allowed for the quantitative detection of O_2_.

Wang et al. reported a ratiometric nanoconjugate for nitric oxide (NO) monitoring, which consisted of UCNP core with dual luminescence emission peaks at 540 and 656 nm, the NO‐sensitive rhodamine B‐derived molecules (RdMs), and the layer of β‐cyclodextrin (βCD) surface ligands.^157^ Here, the reaction of NO with the *O*‐phenylenediamine of RdMs induced the opening of the spiro‐ring and caused the strong absorption of rhodamine B(RdB) at 500–600 nm, which in turn decreased the fluorescence intensity of the UCNPs at 540 nm (Figure 15b). Therefore, the intensity ratio of fluorescence emission at 656 and 540 nm, i.e., the *I*_656_/*I*_540_ ratio, would increase with NO and thus can be used for quantitative sensing for NO. It is interesting to note that an additional β‐CD layer was present on the surface of the particles, which not only improved the biocompatibility but also prevented the RdB from leaching out of the nanoconjugate due to the capping effects. Ali et al. demonstrated a novel sensor for CO_2_ based on the optical interrogation of polystyrene (PS) film containing NaYF_4_:Yb, Er UCNPs, and pH indicator, BTB.^158^ The PS film was selected because it was permeable for CO_2_ but impermeable for protons. The UCNPs generated the green (542 nm) and red (657 nm) emissions when excited with 980 nm laser. When CO_2_ was present, the BTB reacted with CO_2_ and yielded carbonic acid, which was accompanied by the color change of BTB from blue to yellow. Therefore, the intensities of the dual emissions of the UCNPs increased with CO_2_ due to the weaker inner filter effects of the BTB indicator. As the first optical sensor for CO_2_, the response time of 10 s switching from pure Ar to 1% CO_2_ in argon and the detection limit of 0.11% of CO_2_ were achieved using this system.

#### 4.5.3 Temperature Sensor

**Figure 16:**
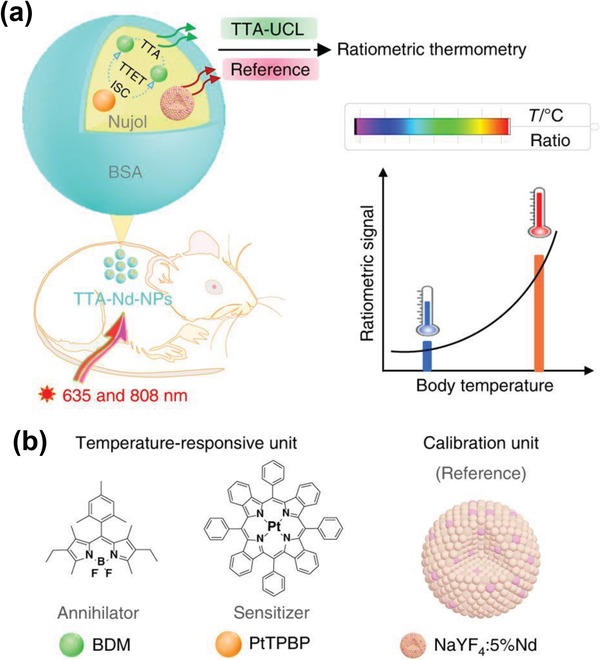
Schematic illustration of the TTA‐Nd‐NPs based ratiometric thermometry in vivo. a) TTA‐upconversion is sensitively responsive to temperature changes. With the assistance of an internal reference, the calibrated TTA–UCL signals become capable for accurate temperature monitoring in a small animal. b) Chemical structures of the TTA chromophores containing BDM (TTA annihilator) and PtTPBP (TTA sensitizer), and schematic structure of the Nd^3+^nanophosphor (reference). BSA: bovine serum albumin, ISC: intersystem crossing, TTET: triplet–triplet energy transfer, TTA: triplet–triplet annihilation. Reproduced with permission.^160^ Copyright 2018, Nature Publishing Group.

Temperature is an essential parameter in biosystems since temperature affects a broad range of intracellular chemical reactions and biological processes.^167^, ^168^Thus, the ability to sense temperature is of great significance in biology and can provide valuable insights into understanding mechanism of various physicochemical processes in living organisms. Vetron et al. devised a nanothermometer based on temperature‐sensitive fluorescence of NaYF_4_:Yb/Er UCNPs, which was capable of accurately measuring the temperature of solutions and HeLa cells.^159^ They reported that the green emission bands of the Er^3+^ emitters (^2^H_11/2_→^4^I_15/2_ and ^4^S_3/2_→^4^I_15/2_) changed with the temperature. Based on this sensing scheme, this fluorescence nanothermometer could measure the internal temperature of living HeLa cell from 25 °C to its death at 45 °C . In a new study, Li and co‐workers developed a highly thermal‐sensitive upconversion system based on the triplet–triplet annihilation (TTA) mechanism (**Figure** **16**a).^160^ A TTA‐upconversion system was formed by coupling the TTA chromophore containing BDM (TTA annihilator) and PtTPBP (TTA sensitizer) with UCNPs (Figure 16b). At high temperature, the TTA‐upconversion was sharply enhanced due to the increase in the diffusion rate and collision probability of chromophores. In addition, an additional thermal‐insensitive internal standard was incorporated into the TTA dyad which allowed for the radiometric thermometry. With this novel TTA‐upconversion system, the ratiometric thermometry in vivo was demonstrated with high thermal sensitivity (≈7.1% K^−1^) and resolution (≈0.1 K), which held great promise for applications in thermometry, nanomedicine, life science, etc.

#### 4.5.4 Metal Ion Sensors

Although the concentration of metal ions such as Ca^2+^, Fe^3+^, Zn^2+^, Cu^2+^, etc., in biological system is not very high, they perform structural and catalytic functions in proteins and enzymes.^169^ Therefore, direct imaging and sensing of metals ions is highly desirable. In this section, the detection of metal ions using UCNPs will be discussed.

**Figure 17:**
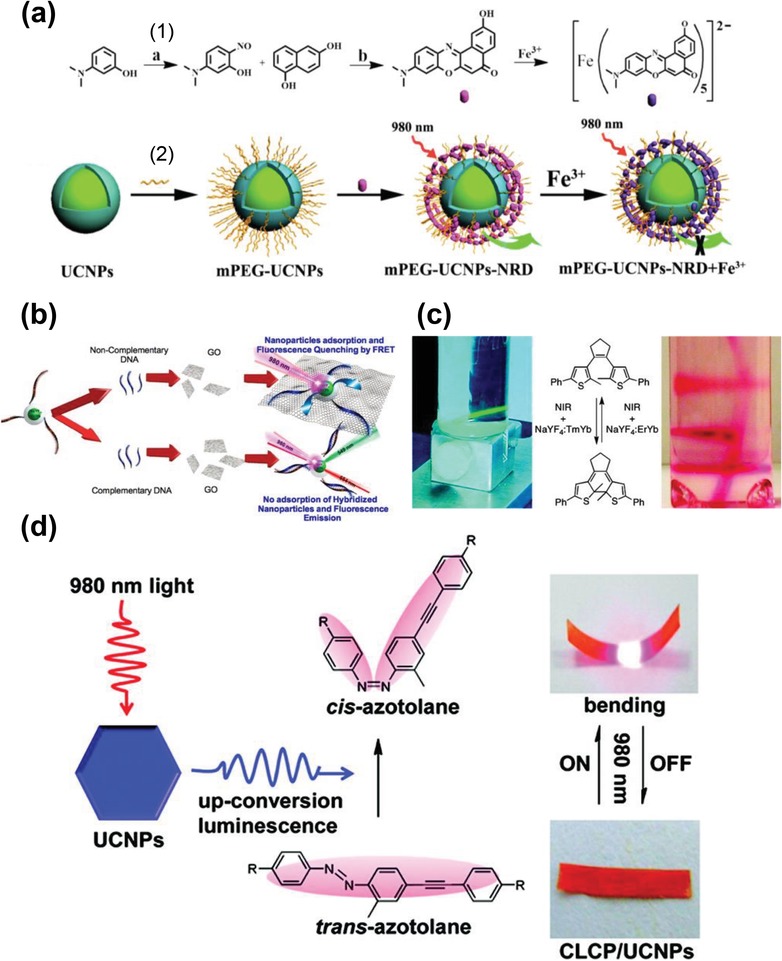
a) Schematic illustration of 1) the synthesis of Nile red derivative (NRD). 2) mPEG‐UCNPs‐NRD and their use for detecting Fe^3+^ based on change in UCL emission. Reproduced with permission.^161^ Copyright 2016, American Chemical Society. b) Schematic representation of the proposed sensor platform under the presence or absence of complementary DNA. Reproduced with permission.^166^ Copyright 2015, American Chemical Society. c) Scheme illustrating the ring‐opening and release reactions of bicyclic compound as it is irradiated with visible or NIR light. Reproduced with permission.^176^ Copyright 2009, American Chemical Society. d) Scheme illustrating UCNP nanophosphors into the azotolane‐containing cross‐linked liquid‐crystal polymer (CLCP) film bending toward the light source along the alignment direction of the mesogens, remaining bent in response to the 980 nm laser irradiation, and becoming flat again after the light source was removed. Reproduced with permission.^179^ Copyright 2011, American Chemical Society.

Fe^3+^ is the most abundant trace element in human body and it is a key element in metabolism of proteins and enzymes involved in various biological processes. Wei et al. synthesized a Fe^3+^‐responsive Nile red derivative (NRD) probe in a conjunction with the NaYF_4_:Yb, Er,Tm@NaGdF_4_ UCNPs, which demonstrated a high selectivity and sensitivity for detecting Fe^3+^ in water and living cells.^161^ The Fe^3+^ addition changed the structure of NRD and increased its absorption, which in turn quenched the emission of UCNPs via the FRET process (**Figure** **17**a). In addition, the Gd doping further increased the effective T_1_ signal, making the nanoprobe also a potential contrast agent for MRI imaging.

Zn^2+^ is the second most abundant trace metal ion in human brain and is required for the catalytic activity of more than 200 enzymes.^170^ Peng et al. developed a rational Zn^2+^ nanosensor by assembling UCNPs with chromophores. They reported that the UCL could be effectively quenched by the chromophores via the FRET process but could again be recovered with the addition of Zn^2+^, which allowed for the quantitative sensing of Zn^2+^.^162^ More importantly, this nanoprobe had also demonstrated an efficient detection of Zn^2+^ in mouse brain slice with Alzheimer's disease (AD) and zebrafish.

Cu^2+^ is the third most abundant trace metal in human body, with a total weight of 75–100 mg.^170^ Although the total amount is rather small, Cu^2+^ is present in every tissue and is essential for maintaining the strength of the skin and blood vessels throughout the body.^170^ Xu et al. constructed a new organic–inorganic hybrid nanoprobe by grafting a rhodamine B derivative (RBH) to the mesoporous SiO_2_ coated UCNPs.^163^ On addition of Cu^2+^, the original green emission of the UCNPs decreased while a new emission band at 580 nm appeared via a FRET process from RBH‐Cu^2+^ to the UCNPs. This novel Cu^2+^ nanoprobe could exclusively detect Cu^2+^ with a detection limit of 0.82 × 10^−6^ m and could be used to monitor subcellular distribution of Cu^2+^ in living cells. Recently, Cui et al found that the aggregation of β‐amyloid (Aβ) protein was closely related to the AD and Cu^2+^ may even promote such aggregation.^164^ Therefore, they designed a nanoprobe combining two components: UCNPs for sensitive detection of Cu^2+^ and the metal chelator, 8‐hydroxyquinoline‐2‐carboxylic acid (HQC), for chelating Cu^2+^ ions. Because of the energy transfer from UCNPs to the Cu^2+^ via the FRET process, this nanoprobe could not only target Cu^2+^‐bonded Aβ_42_ conjugate in zebrafish and AD mice but also achieve the AD treatment by capturing Cu^2+^ from Cu^2+^‐Aβ_42_ conjugate and inhibit Cu^2+^‐induced Aβ_42_ aggregation.

Calcium ion (Ca^2+^) is another trace element that plays vital roles in biochemistry and physiology of cells and organisms. Li et al. proposed a scheme to construct a FRET‐based nanosensor using UCNPs with confined emitters to detect Ca^2+^ in living cells.^165^ The nanosensor had a sandwich structure with the core‐inner shell‐outer shell architecture in which the emitting ions were confined at the inner shell near the particle surface to minimize the donor‐to‐acceptor distance. The specially designed NaYF_4_@NaYF_4_:Yb/Er@NaYF_4_ UCNPs was used as the luminophore and the Ca^2+^ receptor, Fluo‐4, was directly attached on the bared surface of UCNPs. Due to the improved FRET efficiency, the as‐constructed nanoprobe could detect the Ca^2+^ concentration with the detection limit of 15 × 10^−12^ m and broad dynamic range from 15 × 10^−12^ m to 1.35 × 10^−6^ m, which surpassed that of commercial Fluo‐4 Ca^2+^ indicator.

#### 4.5.5 DNA Sensor

DNA is a biopolymer that is arguably the most universal platform for designing, assembling and engineering nanoparticles and nanodevices.^171^ In addition, some DNA or RNA usually appears at the beginning of the disease such that detection of these biomarkers may help to facilitate early diagnostics and reduce the suffering and cost.^172^ One of the common approaches to sense DNA is based on the self‐complementary sequence within a hairpin loop that generates a close contact between the fluorophore and the quencher.^173^, ^174^ With the addition of complementary DNA, the quencher and fluorophore are separated due to the hybridization effect, which turns on the fluorescence of the energy donor and allows for the quantitative sensing for DNA. Due to their unique properties such as less biodamage and weaker auto‐fluorescence background, UCNPs, as the fluorescence donors, have been widely applied to sense and image DNA. For example, the single strand nucleic acids have previously been detected using UCNPs by different groups.^26^, ^175^ Recently, Alonso‐Cristobal et al. demonstrated a DNA nanoprobe using the FRET pair formed by β‐NaYF_4_:Yb/Er UCNPs as the fluorescent donor and graphic oxide (GO) as the quencher.^166^ The single strands of DNA were first attached on the surface of β‐NaYF_4_:Yb/Er@SiO_2_ nanoparticles. Because of the π–π stacking between the GO and DNA, the β‐NaYF_4_:Yb/Er@SiO_2_ nanoparticles were further brought close to the surface of GO and induced a FRET quenching of the fluorescence emission of the NaYF_4_:Yb/Er@SiO_2_ nanoparticles. Upon the addition of complementary DNA strands, the hybridization generated the double‐stranded DNA that did not interact with GO such that the NaYF_4_:Yb/Er@SiO_2_ nanoparticles were separated from its GO quencher and caused the recovery of the fluorescence of UCNPs (Figure 17b). With this novel method, the detection of DNA could be achieved with a detection limit of 5 × 10^−12^ m.

### 4.6 UCNP‐Mediated Chemical Reaction

Photochromic molecules and diarylthene derivatives are sensitive to UV and visible light, generally accompanied with a reversible switch between the ring‐closing and ring‐opening forms.^14^ Carling et al. first demonstrated that the photoswitches of the dithienylethene (DTE) could be triggered by the NIR light.^176^ They synthesized two UCNPs that absorbed 980 nm light, one was NaYF4:Yb/Tm nanoparticles that emitted UV light to induce the ring‐closing reaction, and the other was NaYF_4_:Yb/Er that emitted green light to induce the ring‐opening reactions (Figure 17c). The scheme to trigger the photoswitches of photochromic molecules with the NIR light could greatly overcome detrimental effects using the visible and UV light, such as large damages, undesired side reactions, low penetration into biological tissues, etc.

Photorelease refers to a light‐triggered process in which agents can be released from some “caged” forms using light as the trigger.^177^ Because the light can be easily focused and controlled, the photorelease offers the great potential to provide the “on‐command” delivery to tissues and organisms. Carling et al. once demonstrated a remote photorelease of caged compounds by a NIR‐activated organic–UCNP hybrid system.^177^ In this system, the NaYF_4_:Yb/Tm UCNPs were used to provide UV emission upon 980 nm laser irradiation, which released the carboxylic acid (–COOH) molecules from 3′,5′‐dialkoxybenzoin derivatives. Yang et al. developed a bioimaging system that based on photo‐caged d‐luciferin/UCNPs conjugate.^178^ Upon UV emission of the NaYF_4_:Yb/Tm UCNPs illuminated by 980 nm laser, the d‐luciferin was uncaged and conferred enhanced fluorescence and bioluminescence signals both in vitro and in vivo. Compared with high‐intensity UV or visible light, the application of NIR light enabled a bioimaging in a deep tissue with minimum damages.

Photoisomerization is another type of photo‐triggered process in which some molecules such as azobenzene, undergo a *trans*→*cis* isomerization when irradiated by light with an appropriate wavelength. Wu et al incorporated the UCNP nanophosphors into the azotolane‐containing cross‐linked liquid‐crystal polymer (CLCP) film.^179^ Upon exposure to NIR light at 980 nm, the composite film generated a fast bending due to the *trans*→*cis* photoisomerization of the azotolane units and alignment changes of the mesogens (Figure 17d). More importantly, the bent film could still completely recover to its initial state once the laser light was turned off. Due to the deeper penetration into the tissues and less damage, the NIR light is a more suitable stimulating source in the applications of light‐driven organic actuators in biological systems. Therefore, the NIR‐light‐induced deformable UCNP–CLCP system has great potentials for various biological applications such as artificial muscle‐like actuators and all‐optical switches.^179^

## 5 Conclusion and Future Prospects

In this review, we summarize recent development of rare‐earth doped UCNPs and highlight the achievements in the synthesis, optimization, and various applications. The past few years have witnessed significant progress in synthesizing UCNPs with controllable size, shape, morphology, and phase via various synthetic approaches. The successful synthesis of high quality UCNPs has also broadened the application, ranging from bioimaging, biosensing, cancer treatments to photochemical reactions. Despite these achievements, there are still some limitations to overcome:

1) Up to now, thermolysis and hydro(solvo)thermal methods have become reliable approaches to synthesize rare‐earth doped UCNPs with high crystallinity and uniform size. A great deal of reported UCNPs has relatively large size, ranging from 20 to 100 nm. However, from a point of view of clinical application, functional nanoparticles with ultrasmall size (<10 nm) are more favorable in biosystems as they have several striking advantages such as, less toxicity, easy access to cellular compartment, fast kidney filtration, and rapid excretion from the body.^180^, ^181^ Unfortunately, the smaller size tends to lead the weaker upconversion emission of UCNPs due to the higher surface to volume ratio and fewer sensitizer and emitter ions on the surface.^182^ Currently, there are four strategies to synthesize ultrasmall UCNPs: 1) tuning the amount of OM in the mixture of OA/ODE(oleic acid/1‐octadecence); 2) Ostwald ripening; 3) ion doping; and 4) varying the ratio of Na^+^:RE^3+^:F^−^.^183^ Developing new pathways and designing rational architectures to fabricate smaller and brighter UCNPs may be one of the directions in the future study. For example, Wisser et al. have demonstrated that the emission dye‐sensitization strategy leads to greater quantum yield enhancements for smaller UCNPs,^37^ which may inspire new design of UCNP architecture and open up implementable methods to enhance upconversion performance of ultrasmall UCNPs.
2) It has been well established that the core/shell architecture represents an effective and implementable strategy to significantly increase the emission intensity and quantum yield of UCNPs. In addition, the core/shell structure engineering also allows for the tuning of energy migration pathway and manipulation of interactions between different rare‐earth ions. However, there is still a lack of study on the local structure and chemical organization of core/shell UCNPs. In a very recent study, Hudry et al. investigated the crystallographic and chemical characteristics of ultrasmall core/shell UCNPs (NaGdF_4_:Yb, Er/NaYF_4_) and reported the existence of interdiffusion of the shell element (e.g., Y) into the core.^184^ This study contradicts the common crystallochemical model of core/shell UCNPs and suggests that it is the concentration gradients not the sharp interface that may be present in the core–shell structure. However, in a previous study, Chen et al. reported the structural integrity of core/shell UCNPs (NaYF_4_:Ce/NaYF_4_:Tb) that the Ce and Tb dopant ions are firmly confined in the designed core and shell layers, respectively.^185^ Therefore, more efforts may be necessary to investigate the elemental migrations in the core/shell UCNPs and systematically study the influence of the starting core size, shell growth method and shell thickness on the crystallographic and chemical structure of obtained core/shell UCNPs.
3) Although in vitro and in vivo proofs of concept have been demonstrated for various UCNP‐based theranostic nanoplatforms, very few have been tested or reached the clinical stage. One of the major obstacles to the introduction of UCNPs in clinical use is the long‐term bioeffects on the body. Particularly, considering the complexity of many UCNP‐based theranostic systems, whether these complicated structures can still be stable in the harsh biological environment for a relatively long period still raises a biosafety concern. Therefore, the study of long‐term degradability of UCNPs in biological systems would be necessary. In addition, once these UCNP‐based nanoparticles are taken up, how they function within the body and interact with nervous and immune system still needs to be solved. Therefore, it is critical to clarify the mechanism in which the UCNPs interact with and influence functions of various types of cells.

## Conflict of Interest

The authors declare no conflict of interest.
